# Shared and specific blood biomarkers for multimorbidity

**DOI:** 10.1038/s41591-025-04038-2

**Published:** 2026-01-02

**Authors:** Alice Margherita Ornago, Caterina Gregorio, Federico Triolo, Ann Zenobia Moore, Alessandra Marengoni, Giorgi Beridze, Giulia Grande, Giuseppe Bellelli, Matilda Dale, Claudia Fredolini, Luigi Ferrucci, Laura Fratiglioni, Amaia Calderón-Larrañaga, Davide Liborio Vetrano

**Affiliations:** 1 School of Medicine and Surgery, University of Milano-Bicocca, Milan, Italy; 2 Aging Research Center, Department of Neurobiology, Care Sciences and Society, Karolinska Institutet and Stockholm University, Stockholm, Sweden; 3 Longitudinal Studies Section, Translational Gerontology Branch, National Institute on Aging, Baltimore, MD USA; 4 Department of Clinical and Experimental Sciences, University of Brescia, Brescia, Italy; 5 Stockholm Gerontology Research Center, Stockholm, Sweden; 6 Acute Geriatrics Unit, Fondazione IRCCS San Gerardo dei Tintori, Monza, Italy; 7 Affinity Proteomics Unit Stockholm, Science for Life Laboratory, Department of Protein Science, School of Engineering Sciences in Chemistry, Biotechnology and Health (CBH), Royal Institute of Technology (KTH), Solna, Sweden

**Keywords:** Biomarkers, Diseases

## Abstract

Aging is accompanied by the progressive accumulation of biological deficits, which increases susceptibility to developing multiple chronic diseases (that is, multimorbidity). The biological underpinnings of multimorbidity remain poorly understood. Here we analyzed 54 blood biomarkers reflecting inflammatory, vascular, metabolic and neurodegenerative processes in 2,247 individuals aged 60 and over from the Swedish National Study on Aging and Care in Kungsholmen. Multimorbidity was assessed using three measures: baseline total disease count, baseline multimorbidity patterns identified through latent class analysis and 15-year rate of disease accumulation. Associations between baseline biomarkers and multimorbidity measures were examined using least absolute shrinkage and selection operator regression. Growth differentiation factor 15, hemoglobin A1c, cystatin C, leptin and insulin were consistently and positively associated with all multimorbidity measures. Additional biomarkers demonstrated specific associations with distinct multimorbidity patterns. Moreover, faster disease accumulation was directly associated with gamma-glutamyl transferase and inversely with albumin. Longitudinal results were externally validated in 522 participants from the Baltimore Longitudinal Study of Aging, with comparable predictive accuracy. Our findings suggest that multiple biological processes contribute to multimorbidity through shared and distinct mechanisms. Metabolic disturbances emerged as a key driver of multimorbidity. If confirmed, these processes could represent targets for interventions to mitigate disease accumulation.

## Abstract (web summary)

Growth differentiation factor 15, hemoglobin A1c, cystatin C, leptin and insulin levels were consistently and positively associated with measures of multimorbidity, both cross-sectional and longitudinal, in individuals aged 60 and over.

## Main

Multimorbidity, defined as the co-occurrence of multiple diseases in the same individual, represents an unintended consequence of modern medicine^1^. Several diseases, previously characterized by high mortality rates such as stroke, myocardial infarction or cancer, today can be treated and often turned into chronic conditions that will accompany the affected individual until a postponed death. Up to 90% of people over 60 years of age live with multimorbidity^2–5^. About one third of them will experience only a mild impact on their functioning and quality of life, half will develop complex multimorbidity phenotypes^6^, 10–15% will develop physical frailty^4,7^, 20% will be diagnosed with dementia^8^ and 15–20% will become dependent in activities of daily living^9^. These figures underscore the impact of multimorbidity on functional and quality of life decline in old age, highlighting its relevant personal, clinical and societal implications.

Common multifactorial pathophysiological pathways link aging to the development of multiple diseases in later life. This aligns with the geroscience hypothesis, which posits that cumulative biological deficits and the depletion of physiological resources, driven by the so-called hallmarks of aging (for example, mitochondrial dysfunction, deregulated nutrient sensing, cellular senescence and systemic inflammation) may increase susceptibility to the development of chronic diseases and, consequently, multimorbidity. According to this view, health could be ultimately improved by targeting the biological mechanisms of aging rather than focusing solely on individual diseases^10–12^. Recent studies suggest that these hallmarks of aging could serve as potential targets for future pharmacological interventions aimed at preventing or slowing the onset of multimorbidity^13,14^. However, it is still unclear how these factors operate, interactively or additively, in the development of multimorbidity^15^. Further, previous studies on biomarkers of aging in relation to multimorbidity^16–22^ have been limited to a few biological processes, largely constrained by the biomarker availability in specific cohorts, and have focused on simplistic definitions of multimorbidity (for example, ≥2 co-occurring diseases or a comorbidity index).

Indeed, multimorbidity has been conceptualized in clinically relevant phenotypes beyond disease count. On the one hand, diseases tend to cluster together in nonrandom combinations. Specific multimorbidity patterns, such as cardiometabolic and neuropsychiatric, have been repeatedly described in cohorts of older people^3,7,16,23,24^. These patterns are believed to be at least partially driven by distinct biological mechanisms and have been differentially linked to a variety of outcomes, including dementia, frailty, disability, hospitalization and death^4,7–9,16,25,26^. On the other hand, the rate of accumulation of chronic diseases over time has been suggested as a proxy measure of aging and a meaningful prognostic tool^27^. People who accumulate diseases more rapidly than others are biologically older; in other words, they are aging faster, thereby experiencing earlier functional and cognitive impairments, more intense healthcare utilization and shorter survival^28^.

Despite the growing evidence of the clinical relevance of these multimorbidity measures, little is known about their underlying biological drivers. Beyond environment, lifestyles and healthcare quality^29^, it is likely that specific age-related biological processes may account for the heterogeneous expression of different multimorbidity measures. Therefore, the aim of this study was to identify both shared and unique biological processes underlying different multimorbidity measures, that is, disease patterns and rate of disease accumulation. We leveraged data from the Swedish Study on Aging and Care in Kungsholmen (SNAC-K), a longitudinal population-based cohort with access to multiple baseline blood-based biomarkers reflecting multiple age-related processes, including inflammation, metabolism, neurodegeneration, and vascular and organ damage. By applying dimensionality reduction to 54 biomarkers and linking them to comprehensive clinical data over 15 years, we identified several biomarkers differentially associated with distinct baseline multimorbidity patterns and with accelerated disease accumulation over time, suggesting the existence of both specific and shared biological signatures that underlie different multimorbidity measures. Longitudinal results were externally validated in a sample from the Baltimore Longitudinal Study on Aging (BLSA).

## Results

### Study population characteristics

We analyzed cross-sectional and longitudinal data from 2,247 individuals aged 60 and over, with complete information on 54 baseline blood biomarkers, participating in SNAC-K. SNAC-K includes individuals randomly selected from the population living in a central area of Stockholm who have been thoroughly evaluated by physicians, nurses and neuropsychologists. The mean age of participants was 72.7 years (s.d. 10.7 years) and 1,383 (61.5%) were females. Table 1 shows the baseline study population characteristics by number of chronic diseases. Participants in the study, at baseline, had an average of 3.9 (s.d. 2.4) chronic diseases, were taking a mean of 3.7 (s.d. 3.3) medications and had an average Mini-Mental State Examination (MMSE) score of 28.3 (s.d. 2.9). In addition, impairment in at least one activity of daily living was present in 4.3% of the population. Individuals with a higher number of chronic diseases were older, more likely to be female and exhibited poorer socioeconomic, clinical and functional profiles.

**Table 1:** Population characteristics at baseline by number of chronic diseases

Baseline characteristics	Overall 2,247	0 to 1 chronic diseases 337 (15%)	2 to 3 chronic diseases 823 (36.6%)	4 to 5 chronic diseases 591 (26.3%)	6+ chronic diseases 496 (22.1%)
Age, mean (s.d.)	72.71 (10.7)	64.68 (6.5)	69.48 (9.1)	74.51 (10.1)	81.37 (9.4)
Women, *n* (%)	1,383 (61.5)	179 (53.1)	489 (59.4)	384 (65.0)	331 (66.7)
BMI, mean (s.d.)	25.66 (4.1)	24.71 (2.8)	25.56 (3.6)	26.13 (4.4)	25.94 (5.1)
Education: high school or above, *n* (%)	1,894 (84.3)	314 (93.2)	715 (86.9)	487 (82.4)	378 (76.2)
Partnered, *n* (%)	1,091 (48.6)	223 (66.2)	445 (54.2)	266 (45.0)	157 (31.7)
Institutionalized, *n* (%)	37 (1.6)	0 (0.0)	2 (0.2)	5 (0.8)	30 (6.0)
**Clinical and functional status**					
1+ activity of daily living lost, *n* (%)	96 (4.3)	1 (0.3)	9 (1.1)	22 (3.7)	64 (12.9)
MMSE score, mean (s.d.)	28.29 (2.9)	29.13 (1.9)	28.87 (1.8)	28.28 (2.4)	26.79 (4.5)
Active physical level, *n* (%)	1,589 (70.7)	274 (81.3)	645 (78.4)	430 (72.8)	240 (48.4)
Number of chronic diseases, mean (s.d.)	3.85 (2.4)	0.75 (0.4)	2.52 (0.5)	4.46 (0.5)	7.45 (1.7)
Number of drugs, mean (s.d.)	3.67 (3.3)	1.18 (1.6)	2.29 (2.1)	4.35 (2.8)	6.83 (3.6)

A mismatch between total numbers and sample sizes is due to missing data. Missing values for civil status,  2 for 2 to 3 chronic diseases; 1 for 6 or more chronic diseases; BMI,  1 for 0 or 1 chronic diseases; 17 for 2 to 3 chronic diseases; 23 for 4 to 5 chronic diseases; 55 for 6 or more chronic diseases; MMSE,  2 for 0 or 1 chronic diseases; 1 for 2 to 3 chronic diseases; 4 for 4 to 5 chronic diseases; 1 for 6 or more chronic diseases. BMI, body mass index.

### Five distinct multimorbidity patterns were identified

**Fig. 1:**
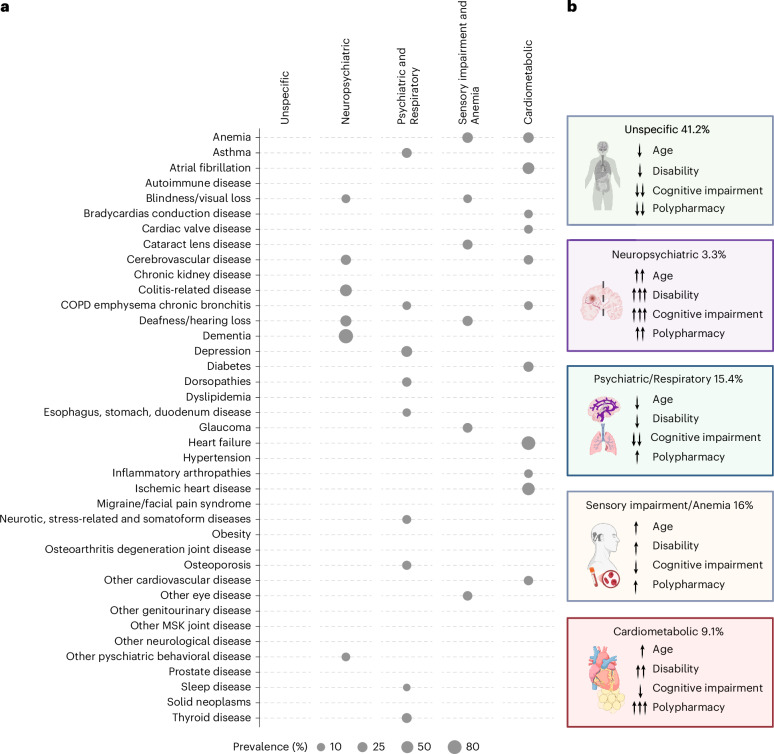
Bubble plot of overexpressed diseases across the five multimorbidity patterns identified and main baseline characteristics by pattern. Summary of the results of the LCA through which five homogeneous patterns (that is, combinations) of chronic diseases were identified. Although the majority of diseases were present across all multimorbidity patterns, each pattern was named after the most overrepresented diseases. **a**, Bubble plot summarizing the diseases overexpressed across the five multimorbidity patterns. Diseases were deemed as overexpressed if they displayed an O/E ratio of at least 2 and an exclusivity of at least 25%. **b**, Main baseline characteristics of participants across the five multimorbidity patterns. Note: arrows are visual markers for descriptive illustration of participants’ baseline characteristics. They denote statistically significant differences across multimorbidity patterns based on Benjamini-Hochberg-adjusted pairwise comparisons. Arrow direction indicates whether a characteristic is elevated or reduced relative to other patterns. The number of arrows visually ranks patterns across the full spectrum of each characteristic—higher numbers of arrows reflect a more extreme position. Patterns with identical arrows—both in number and direction—do not differ significantly for that characteristic. Illustrations in **b** created using BioRender.com. COPD, chronic obstructive pulmonary disease; MSK, muscle skeletal.

In SNAC-K, 60 categories of chronic diseases^3^ were ascertained through clinical examination, anamnesis, instrumental tests, laboratory tests, self-reported medication use and electronic health records. Homogeneous groups of participants with multimorbidity (that is, two or more diseases) sharing similar patterns of chronic diseases were identified at baseline through latent class analysis (LCA) and the overexpressed diseases, defined by indexes of observed/expected (O/E) ratio and exclusivity, were used to label the patterns. The overexpressed diseases across the five patterns are shown in Fig. 1a by means of a bubble plot. Participants were assigned to the pattern for which they had the highest probability of membership; notably, they may still exhibit additional chronic conditions that were not overexpressed in their assigned pattern. At baseline, the study population was classified into six groups, one group of participants with zero or one disease (that is, no multimorbidity, comprising 337 (15%) individuals), and five homogeneous patterns of multimorbidity identified via LCA: Unspecific (that is, no disease overexpressed; prevalence of 41.2%); Neuropsychiatric (3.3%); Psychiatric and Respiratory (15.4%); Sensory impairment and Anemia (16%) and Cardiometabolic (9.1%). Baseline sample characteristics across the six groups are reported in Supplementary Table 1. In Fig. 1b, we summarize the main sociodemographic, clinical and functional characteristics of participants across multimorbidity patterns. The Unspecific pattern included the youngest, least dependent participants with the lowest cognitive impairment and polypharmacy. The Neuropsychiatric pattern was characterized by older participants with greater physical disability, higher polypharmacy and more marked cognitive impairment. The Cardiometabolic pattern showed moderate functional impairment and high polypharmacy. Participants in the Sensory impairment and Anemia pattern had mild disability, moderate polypharmacy and less cognitive impairment. Finally, the Psychiatric and Respiratory pattern included younger participants with low disability and cognitive impairment but moderate polypharmacy.

In relation to distal outcomes (Supplementary Table 2), the Neuropsychiatric pattern demonstrated a higher probability of incident dementia and risk of recurrent depressive episodes. A higher risk of these depressive episodes was also observed in the Psychiatric and Respiratory pattern. The Cardiometabolic pattern was associated with a higher probability of developing heart failure and ischemic heart diseases. Additionally, 15-year all-cause mortality rates were higher in the Neuropsychiatric, the Cardiometabolic and the Psychiatric and Respiratory patterns compared to the No-multimorbidity group (Supplementary Table 3).

### Association between biomarkers and cross-sectional measures of multimorbidity

In the present study, 54 blood biomarkers were available in SNAC-K at baseline, and their association with different multimorbidity measures was tested (Methods). The complete list of biomarkers is reported in Supplementary Tables 4 and5.

**Extended Data Fig. 1:**
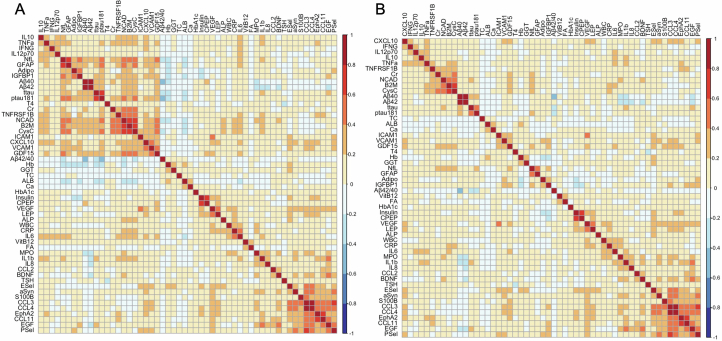
Correlation matrix of SNAC-K blood-based biomarkers. **Panel A**) Unadjusted Spearman correlation between blood-based biomarkers. **Panel B**) Age-adjusted Spearman correlation between blood-based biomarkers. Aβ 40 = amyloid beta protein 40; Aβ 42 = amyloid beta protein 42; Adipo = adiponectin; ALB = albumin; ALP = alkaline phosphatase; aSyn = alpha synuclein; BDNF = Brain-derived neurotrophic factor; B2M = beta-2-microglobulin; Ca = calcium; CCL11 = C-C motif chemokine 11; CCL2 = C-C motif chemokine 2; CCL3 = C-C motif chemokine 3; CCL4 = C-C motif chemokine 11; CPEP = C-peptide; Cr = creatinine; CRP = C-reactive protein; CXCL10 = chemokine CXC ligand 10; CysC = cystatin C; EGF = Epidermal growth factor; EphA2 = ephrin type-A receptor 2; ESel = E-selectin; FA = folic acid; GDF15 = growth differentiation factor 15; GFAP = Glial fibrillary acidic protein; GGT = gamma-glutamyl transferase; Hb = hemoglobin; HbA1c = hemoglobin A1c; ICAM1 = Intercellular Adhesion Molecule 1; IFNG = Interferon gamma; IGFBP1 = Insulin-like growth factor binding protein 1; IL-1b = interleukin 1 beta; IL-10 = interleukin 10; IL-12p70 = Interleukin 12p70; IL-6 = interleukin 6; IL-8 = interleukin 8; LEP = leptin; MPO = Myeloperoxidase; NCAD = N-Cadherin; NfL = neurofilament light chain; PSel = P-selectin; p-tau181 = phosphorylated tau 181; S100B = S100 calcium-binding protein B; T4 = Thyroxine; TC = total cholesterol; TNFa = tumor necrosis factor alpha; TNFRSF1B = tumor necrosis factor receptor superfamily member 1B; TSH = thyroid stimulating hormone; VCAM1 = vascular cell adhesion protein 1; VEGF = vascular endothelial growth factor; VitB12 = vitamin B12; WBC = leucocytes.

To identify the set of biomarkers best for predicting the baseline number of chronic diseases while effectively accounting for multicollinearity (see correlation matrix in Extended Data Fig. 1), a Gaussian least absolute shrinkage and selection operator (LASSO) analysis, adjusted for age, sex and education, was performed. Eight biomarkers were found to be independently and directly associated with the baseline number of chronic diseases (that is, cystatin C, hemoglobin A1c (HbA1c), growth differentiation factor 15 (GDF15), leptin, insulin, neurofilament light chain (NfL), creatinine and C-peptide), while hemoglobin was reversely associated with this multimorbidity measure.

**Fig. 2:**
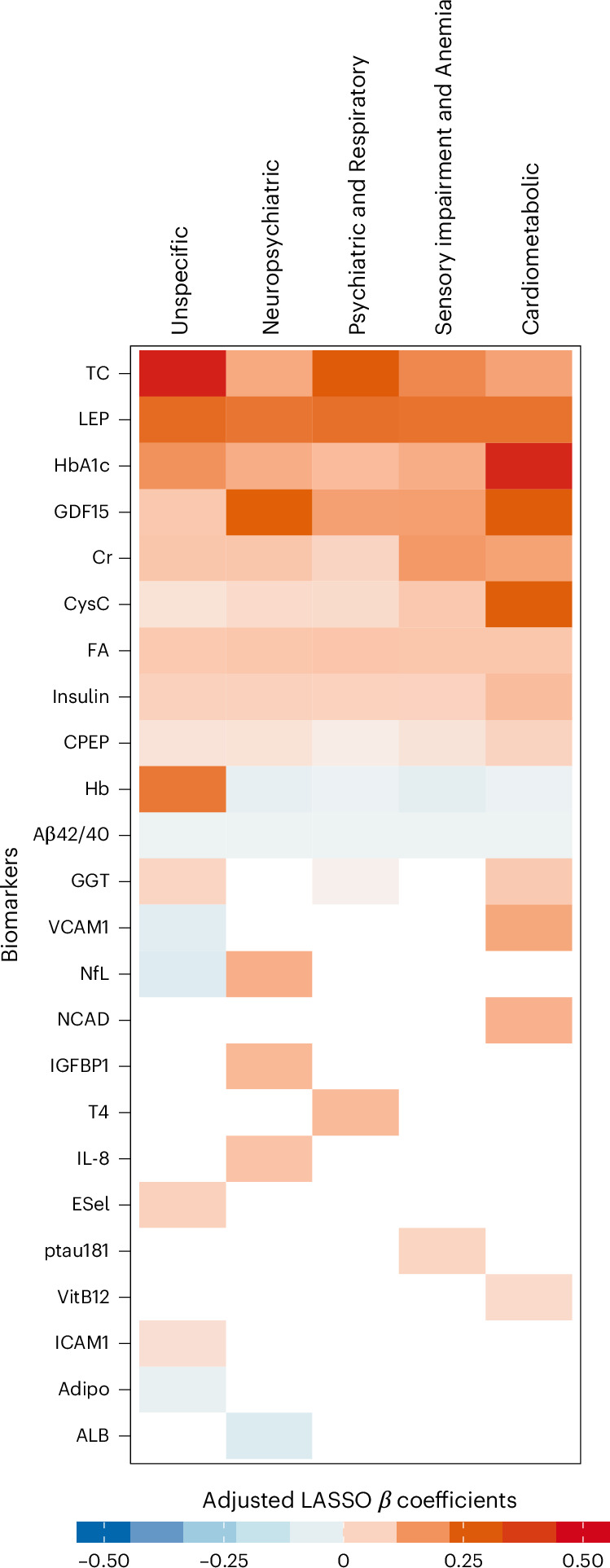
Heatmap of adjusted multinomial LASSO *β* coefficients of biomarkers associated with multimorbidity patterns. Heatmap showing the mean *β* coefficients from 1,000 multinomial LASSO regression models, adjusted for age, sex and education. The *β* coefficients were derived using the No-multimorbidity (that is, zero or one disease) group as the reference category. The absolute value of the *β* coefficient reflects the strength of the association, while the sign of the *β* coefficient indicates whether the association is direct or reverse. *β* coefficients equal to zero (that is, white) indicate that the corresponding biomarker has no significant effect on the dependent variable when considering the influence of all other biomarkers in the model. Aβ40, amyloid β protein 40; Aβ42, amyloid β protein 42; Adipo, adiponectin; ALB, albumin; CPEP, C-peptide; Cr, creatinine; CysC, cystatin C; ESel, E-selectin; FA, folic acid; GGT, gamma-glutamyl transferase; Hb, hemoglobin; ICAM1, intercellular adhesion molecule 1; IGFBP1, insulin-like growth factor binding protein 1; IL-8, interleukin 8; LEP, leptin; NCAD, N-Cadherin; ptau181, phosphorylated tau 181; T4, thyroxine; TC, total cholesterol; VCAM1, vascular cell adhesion protein 1; VitB12, vitamin B12.

**Extended Data Fig. 2:**
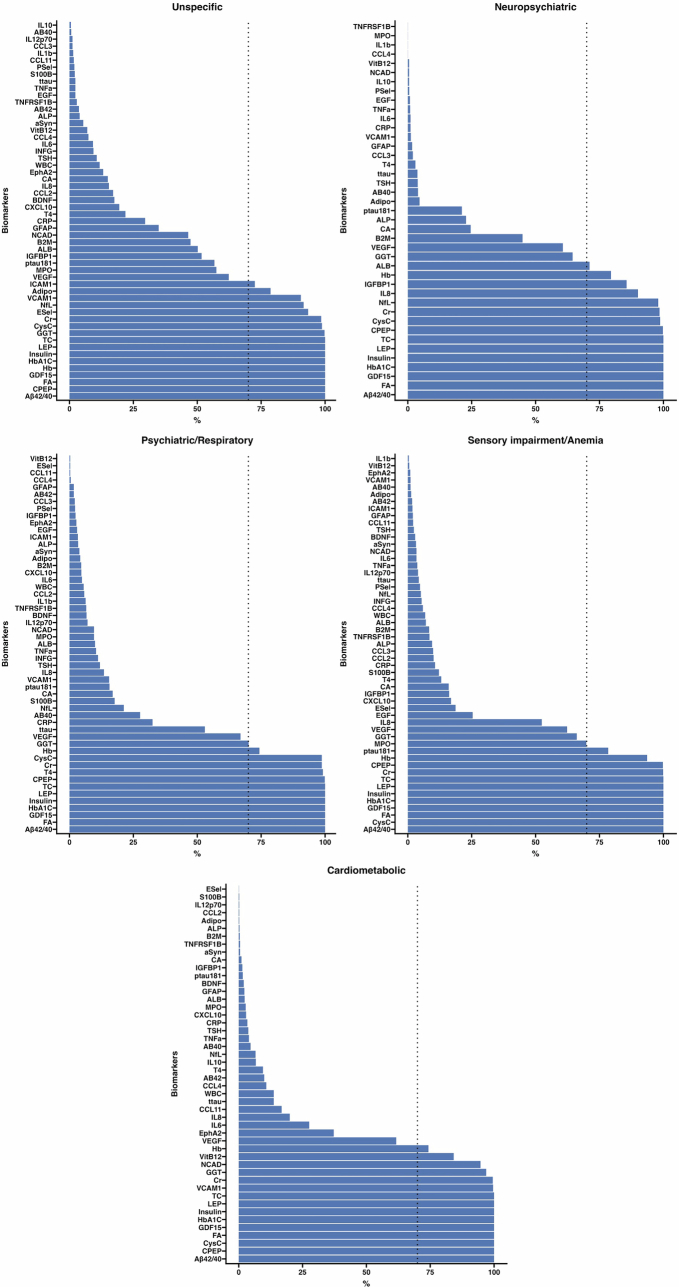
Barr plots of the biomarkers with non-zero coefficients in the 1000 multinomial LASSO models. Each Barr plot shows the biomarkers selected by the 1000 multinomial LASSO models. Barr height represents the percentage of time a biomarker was selected for the given multimorbidity pattern. The dotted line indicates the 70% selection threshold. Aβ 40 = amyloid beta protein 40; Aβ 42 = amyloid beta protein 42; Adipo = adiponectin; ALB = albumin; ALP = alkaline phosphatase; aSyn = alpha synuclein; BDNF = Brain-derived neurotrophic factor; B2M = beta-2-microglobulin; Ca = calcium; CCL11 = C-C motif chemokine 11; CCL2 = C-C motif chemokine 2; CCL3 = C-C motif chemokine 3; CCL4 = C-C motif chemokine 11; CPEP = C-peptide; Cr = creatinine; CRP = C-reactive protein; CXCL10 = chemokine CXC ligand 10; CysC = cystatin C; EGF = Epidermal growth factor; EphA2 = ephrin type-A receptor 2; ESel = E-selectin; FA = folic acid; GDF15 = growth differentiation factor 15; GFAP = Glial fibrillary acidic protein; GGT = gamma-glutamyl transferase; Hb = hemoglobin; HbA1c = hemoglobin A1c; ICAM1 = Intercellular Adhesion Molecule 1; IFNG = Interferon gamma; IGFBP1 = Insulin-like growth factor binding protein 1; IL-1b = interleukin 1 beta; IL-10 = interleukin 10; IL-12p70 = Interleukin 12p70; IL-6 = interleukin 6; IL-8 = interleukin 8; LEP = leptin; MPO = Myeloperoxidase; NCAD = N-Cadherin; NfL = neurofilament light chain; PSel = P-selectin; p-tau181 = phosphorylated tau 181; S100B = S100 calcium-binding protein B; T4 = Thyroxine; TC = total cholesterol; TNFa = tumor necrosis factor alpha; TNFRSF1B = tumor necrosis factor receptor superfamily member 1B; TSH = thyroid stimulating hormone; VCAM1 = vascular cell adhesion protein 1; VEGF = vascular endothelial growth factor; VitB12 = vitamin B12; WBC = leucocytes.

In addition, an adjusted multinomial LASSO analysis was employed to select a set of biomarkers that most strongly influence the odds of belonging to each multimorbidity pattern as defined at baseline, as compared to the No-multimorbidity group. Multinomial LASSO was performed across 1,000 resampled multimorbidity pattern membership datasets derived from the LCA models, retaining those biomarkers selected in at least 70% of the models as repeatedly associated with multimorbidity patterns (Extended Data Fig. 2). The results of the LASSO selection are summarized in Fig. 2. The heatmap enables the comparison of the intensity and direction of the associations between blood biomarkers and different multimorbidity patterns. Two different types of biomarker behaviors were observed: some biomarkers were associated—either directly or reversely—with all multimorbidity patterns, while others showed more pattern-specific associations. The following biomarkers were directly associated with all multimorbidity patterns: C-peptide, creatinine (especially with the Sensory impairment and Anemia and the Cardiometabolic patterns), cystatin C (especially with the Cardiometabolic pattern), GDF15 (especially with the Neuropsychiatric pattern and the Cardiometabolic pattern), folic acid, HbA1c (especially with the Cardiometabolic pattern), insulin, leptin and total cholesterol (especially with the Unspecific pattern and the Psychiatric and Respiratory pattern). Conversely, the amyloid β 42/40 ratio was reversely associated with all multimorbidity patterns, while hemoglobin showed a reverse association with all patterns, except with the Unspecific pattern, with which it was strongly and directly associated. Finally, as shown in Fig. 2, several biomarkers were found to be specific to one or two multimorbidity patterns.

### Association between biomarkers and the rate of accumulation of chronic diseases

**Fig. 3:**
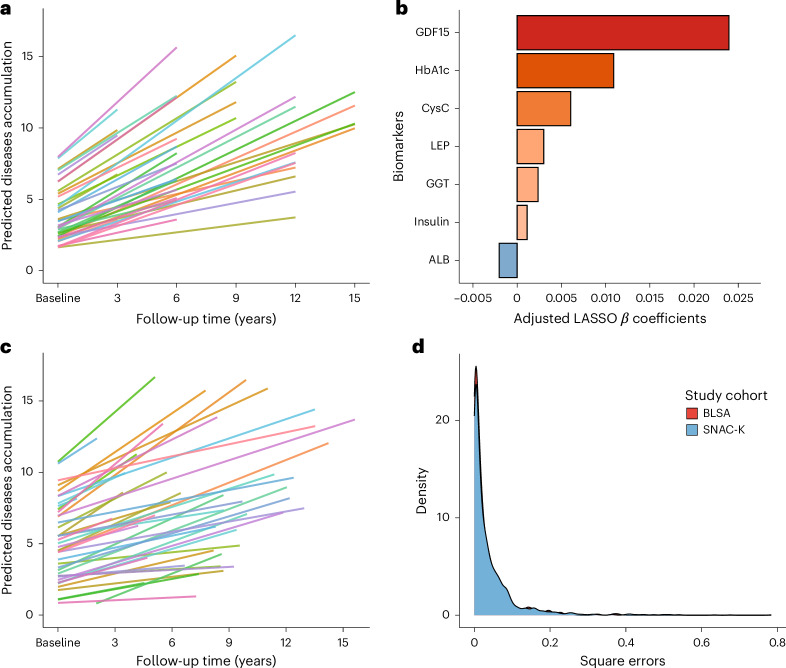
Trajectories of disease accumulation and biomarkers associated with an accelerated multimorbidity progression. **a**, Longitudinal trajectories of chronic disease accumulation modeled using a linear mixed model. Each trajectory represents the change in the number of chronic diseases over 15 years for 50 randomly selected participants from the SNAC-K study population. **b**, Standardized *β* coefficients obtained from a Gaussian LASSO regression model adjusted for age, sex and education. **c**, Longitudinal trajectories of disease accumulation in the BLSA cohort, modeled with linear mixed model, based on 50 randomly selected participants. **d**, Comparisons of square errors distributions between the study populations of SNAC-K and BLSA. MSE: SNAC-K = 0.041, BLSA = 0.032.

The association between baseline biomarkers and the rate of accumulation of chronic diseases over 15 years of follow-up was assessed using linear mixed models adjusted by age, sex and education. A Gaussian LASSO model was employed to identify biomarkers independently associated with a faster accumulation of chronic diseases (that is, β coefficients of the slope derived from the linear mixed model). GDF15, HbA1c, cystatin C, leptin, gamma-glutamyl transferase and insulin were directly associated with accelerated multimorbidity development, while albumin was reversely associated with the rate of disease accumulation (Fig. 3b).

### Principal component analysis of LASSO-selected biomarkers

**Fig. 4:**
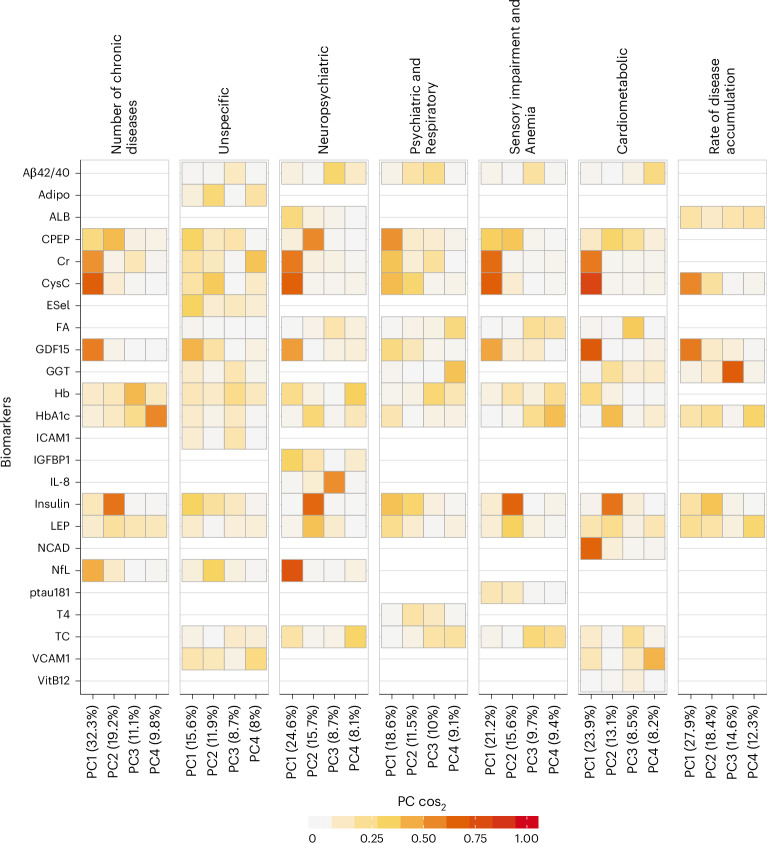
PCA of LASSO-selected biomarkers for disease number and multimorbidity patterns at baseline, and rate of disease accumulation during follow-up. The individual contributions of each principal component on the different biomarkers are quantified through ${\cos }^{2}$. A high ${\cos }^{2}$ value indicates a great importance of that component for the biomarker.

To identify specific baseline biomarker subprofiles or combinations among those that were significantly associated with multimorbidity measures, principal component analysis (PCA) was carried out within each multimorbidity measure. The contributions of biomarkers to the first four principal components are depicted through a heatmap in Fig. 4. Different biomarkers were found to be overexpressed across different principal components (PCs) and multimorbidity measures (Supplementary Table 6).

GDF15 was constantly represented in PC1 across all multimorbidity measures except the Psychiatric and Respiratory pattern, whereas cystatin C was represented in PC1 across all patterns except the Unspecific pattern. Of note, PC1 explained 15–32% of the variance across different multimorbidity measures. However, the specific biomarker subprofiles associated with PC1 varied slightly across the multimorbidity measures. In the Neuropsychiatric pattern and the number of chronic diseases, PC1 was characterized by high contributions from creatinine and NfL. The Sensory impairment and Anemia pattern showed a high contribution of creatinine in PC1, while the Cardiometabolic pattern showed strong contributions from both creatinine and N-cadherin. A secondary main trend driven by C-peptide and insulin was observed in the number of chronic diseases, the Neuropsychiatric pattern, the Sensory impairment and Anemia pattern and the Cardiometabolic pattern. The rate of chronic disease accumulation showed a high contribution of insulin in PC2, and a prominent contribution of gamma-glutamyl transferase in PC3. While most multimorbidity measures showed a cumulative explained variance above 50% across the first four principal components, the Psychiatric and Respiratory and the Unspecific patterns exhibited a notably lower explained variance, with the latter lacking any clear underlying structure.

### External validation of the longitudinal findings

**Extended Data Fig. 3:**
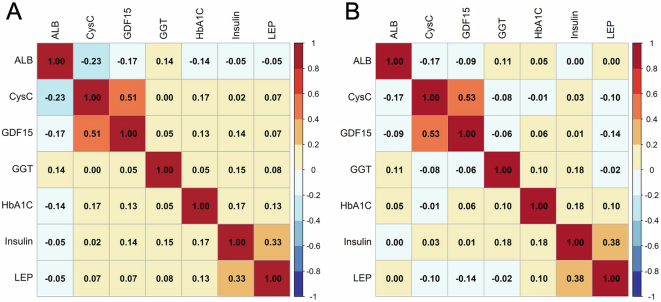
Correlation matrix of LASSO-selected biomarkers: SNAC-K vs. **BLSA Panel A**) correlation plots of biomarkers in SNAC-K cohort. **Panel B**) correlation plots of biomarkers in BLSA cohort. ALB = albumin; CysC = cystatin C; GDF15 = growth differentiation factor 15; GGT = gamma-glutamyl transferase; HbA1c = hemoglobin A1c; LEP = leptin.

To validate our longitudinal results externally, we evaluated the predictive performance of the LASSO-selected biomarkers associated with the rate of disease accumulation over time in a different cohort. We used data from 522 participants (mean age: 75.7 years; 51.7% female) of the BLSA (Supplementary Table 7). To assess the comparability of biomarker profiles, we examined Spearman correlations among the LASSO-selected biomarkers across the two cohorts. The correlations were highly similar (Extended Data Fig. 3), indicating that the interrelationships between biomarkers were similar despite differences in cohort composition. A linear mixed-effect model was used to estimate the individual rate of disease accumulation (Fig. 4c). Then, the SNAC-K LASSO-derived coefficients were applied to the BLSA data to test the model’s ability to generalize in terms of average prediction error. The mean square error (MSE) between predicted and observed slopes in the BLSA cohort was 0.032, compared to the MSE of 0.041 in the SNAC-K cohort (Fig. 4d). This suggests that the model generalizes well and is not overfitted to the training data. Given that the outcome reflects the annual rate of disease accumulation, the square root of the MSE corresponds to an average prediction error of approximately 0.18 diseases per year in BLSA and 0.19 diseases per year in SNAC-K, indicating a highly consistent and clinically meaningful level of accuracy across cohorts.

**Fig. 5:**
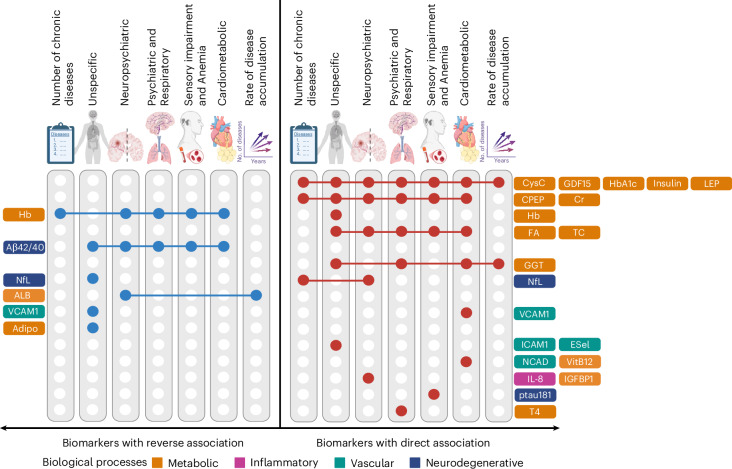
Summary of the shared and specific biomarkers across multimorbidity measures identified through LASSO analysis. An illustratration of LASSO-selected shared and specific biomarkers across the different multimorbidity measures operationalized in the study. Blue dots represent biomarkers with a reverse association with the multimorbidity measure depicted at the top of the columns, while red dots indicate those with a direct association. Biomarkers are categorized according to the biological process they are associated with. Illustrations created using BioRender.com.

Fig. 5 provides a comprehensive summary of the LASSO-selected shared and specific baseline biomarkers across multimorbidity measures.

## Discussion

In this study we analyzed the association between a range of blood biomarkers that describe diverse biological processes—metabolic, inflammatory, vascular, organ damage and neurodegenerative—with several multimorbidity measures. Three major findings emerged from the analysis, all consistently pointing to the major role played by metabolic dysregulation involving glucose and energy control pathways in the expression and progression of multimorbidity. First, regardless of how multimorbidity was operationalized, several biomarkers were consistently directly associated with multimorbidity: GDF15, HbA1c, cystatin C, leptin and insulin. In addition to these, gamma-glutamyl transferase and albumin (inversely) were also associated with a faster expansion of multimorbidity over time. Second, some biomarkers appeared to be specific to individual or few multimorbidity patterns, albeit with different intensities and directions of association. Finally, by further dissecting through PCA the biomarkers associated with the different multimorbidity measures, we identified and quantified mainstream biological subprofiles, which could raise interest as potential targets of interventions. Of note, our longitudinal findings were successfully validated in an external and independent cohort.

In previous research, the biomarkers most frequently found to be associated with the presence of multiple diseases were primarily inflammatory (for example, IL-6 and C-reactive protein), metabolic (for example, HbA1c, GDF15 and cystatin C) and neurodegenerative (for example, amyloid β)^16–22^. However, these studies, in addition to the diverse and heterogeneous operationalization of multimorbidity, are often limited by the cross-sectional design and the use of biomarkers proxying only one or a few biological domains. The strength of our study lies in the use of several multimorbidity measures including the longitudinal perspective, the use of a wide range of biomarkers proxying diverse biological processes and also in the application of advanced analytical techniques that allowed us to account for biomarkers’ interdependence.

Through this approach, our study identified five biomarkers directly associated with and shared across multimorbidity measures. Of note, these biomarkers have been previously recognized within the framework of blood biomarkers for geroscience^30^. GDF15, a senescence-associated protein within the TGF-β superfamily, is a biomarker of aging related to mitochondrial dysfunction and systemic stress response, including oxidative stress and metabolic disturbances^31^. GDF15 consistently presented with the strongest effect sizes in relation to multimorbidity, suggesting either a strong mechanistic role of this biomarker or its pleiotropic downhill response to several other biological processes and signaling pathways, including its involvement in the integrated stress response, where it may modulate inflammation and contribute to adaptive resilience under conditions of cellular distress^31^. Cystatin C, an extracellular inhibitor of cysteine proteases, is a marker of renal function as well as aging and has been proposed as a potential prognostic marker in various disease states associated with inflammation, oxidative stress and endothelial dysfunction, such as various cardiovascular diseases and metabolic disorders^32^. This finding points out the possible important role played by renal health in accelerating the aging process. Lastly, insulin, leptin and HbA1c reflect glucose and fat metabolism, food intake control and energy homeostasis, and serve as markers of metabolic aging^33,34^. Taken together, these biomarkers are involved in a cycle of metabolic and inflammatory dysregulation that could underlie the pathogenesis of multimorbidity. It is increasingly evident that cellular metabolism influences the immune system and contributes to signaling pathways associated with and responsible for the development of multiple diseases^35^. Metabolic status is a key determinant of immune function, which, in turn, plays a pleiotropic role in metabolism to maintain organismal homeostasis^36^. However, when these systems become imbalanced, low-grade systemic inflammation perpetuates metabolic alterations by establishing a cycle that leads to pathological processes such as insulin resistance, atherosclerosis and endothelial dysfunction that underlie a variety of diseases^37–39^. Our findings suggest that metabolic dysregulation may represent a central signature in the development of multimorbidity, as reflected by the consistent association of metabolic biomarkers across all multimorbidity measures. Notably, these biomarkers are not solely metabolic but also reflect chronic inflammatory and immunometabolic stress (for example, oxidative stress, impaired mitochondrial health and immunoendocrine signaling). Their dual role may explain their stronger and more consistent association with multimorbidity, highlighting the centrality of immunometabolic disturbances in the accumulation of several chronic diseases. Therefore, our finding suggests that a shared pathophysiological mechanism closely linked to metabolic disturbances may underlie the development of multimorbidity. The exceptionally high signal found for GDF15 might suggest this as a reliable biomarker of complex and expanding multimorbidity.

By looking into distinct disease patterns, the contribution of metabolic disturbances becomes increasingly evident. In particular, C-peptide, a marker of pancreatic function and glucose metabolism^40^, folic acid and total cholesterol, both markers of nutritional status, and creatinine, a marker of renal function and muscle health^41^, emerged as directly associated with the five multimorbidity patterns. Furthermore, hemoglobin—which is strongly impacted by nutritional status and responds to inflammation in old age—was reversely associated with all multimorbidity patterns, except the Unspecific pattern. Finally, the accumulation rate of chronic disease, along with the broadly shared mechanism described above, was strongly associated with gamma-glutamyl transferase, a marker of liver function linked to systemic inflammation, and reversely with albumin, a marker of nutritional status, liver function and overall health. Interestingly, a reverse association emerged between the amyloid β 42/40 ratio and all multimorbidity patterns. Previous studies suggest that cardiovascular and cerebrovascular factors may influence the peripheral production and metabolism of amyloid markers^42^. Therefore, the consistency of this association across all multimorbidity patterns—rather than its specificity to the Neuropsychiatric pattern—is not unexpected, as blood-based amyloid β levels reflect production from both the central nervous system and, more prominently, peripheral tissues^43^. In contrast, NfL, a neuron-specific cytoskeletal component, showed a more selective and direct association with the Neuropsychiatric pattern, consistent with its origin in axonal damage. Methodological factors related to amyloid β measurements should also be considered, as alternative analytical techniques demonstrated greater robustness compared to the assay used in our study^44,45^. These considerations, along with a recent review reporting inconsistent associations between the amyloid β 42/40 ratio and multimorbidity^46^, underscore the need for cautious interpretation and further investigation.

In addition to these common biological processes, more specific biomarkers were found to be associated with the distinct clustering of diseases, with some emerging as important contributors to subprofiles identified through PCA. NfL, linked to cognitive function in previous studies^47–49^, was strongly and directly associated with the primary trend in the Neuropsychiatric pattern. N-cadherin, a protein involved in cardiomyocyte structural integrity^50^, exhibited a strong direct association with the main principal component in the Cardiometabolic pattern. In contrast, the Unspecific pattern did not exhibit unique biomarker subprofiles, possibly explained by the fact that the diseases mostly represented in this pattern are largely responsive to the mainstream and more generic aging and multimorbidity-related processes underlined above.

Taken together, these findings reinforce the geroscience funding hypothesis, which posits that genetic, molecular and cellular factors underlying biological aging as a major risk factor and driver of common chronic conditions and diseases, thus predisposing the individual with a given biological profile to a more rapid development of multimorbidity and consequently also of frailty, disability and mortality^14,51^. At the same time, our findings identify more specific pathways that may serve to uncover biological processes that deviate or synergistically contribute to the geroscience hypothesis.

The primary subprofile most strongly associated with multimorbidity as identified through PCA, both cross-sectionally and longitudinally, involved GDF15 and cystatin C, key markers of mitochondrial and renal dysfunction, respectively, both of which promptly respond to stress and metabolic disturbances. Additionally, a secondary subprofile identified through the longitudinal analysis highlighted the contribution of metabolic processes encompassing insulin, HbA1c and gamma-glutamyl transferase. Together, these findings further emphasize shared pathological mechanisms, rooted in a cycle of metabolic, immunometabolic and inflammatory dysregulation, driving the development and progression of multimorbidity.

These findings carry considerable clinical and research relevance. First, the identified blood biomarkers demonstrate prognostic value by aiding in the identification of specific biological processes, which could pave the way for advancing our understanding of the shared and specific mechanisms driving given patterns of diseases and developing targeted interventions in the future. While the pathophysiological processes underlying individual diseases are often well established, the mechanisms underpinning multimorbidity remain less understood. Investigating these mechanisms is crucial, as complex combinations of diseases frequently result in adverse outcomes driven by their interactions. This underscores the importance of not only characterizing the clinical measures of multimorbidity but also untangling the biological phenotypes that form the foundation of these associations. In this context, the centrality of metabolic disturbances in the process of multimorbidity underscores the importance of maintaining an active and healthy lifestyle as a cornerstone of chronic disease prevention and management. Emerging evidence also suggests that certain antidiabetic agents may exert pleiotropic benefits beyond glycemic control, including cardiovascular, renal, neuroprotective, musculoskeletal and anti-inflammatory effects, even in nondiabetic populations^52–55^. While lifestyle interventions remain indispensable, these preliminary findings warrant further investigation to elucidate the broader therapeutic potential of these pharmacological strategies and to identify the population most likely to benefit.

This study has some limitations. Individuals who participated in SNAC-K are comparatively healthier, with a higher socioeconomic status and are primarily of Swedish origin. This may limit the generalizability of the findings. Although the 54 biomarkers were selected through expert consensus and extensive literature review, the panel may not fully capture the biological complexity underlying disease development and may have overlooked emerging or understudied markers. Nonetheless, our aim was to reliably characterize ongoing biological processes, rather than to identify specific biomarkers for multimorbidity itself. The use of serum instead of plasma in the assays may have contributed to lower concentrations of the measured proteins. In addition, in cross-sectional analyses, it is not possible to determine whether the biomarkers reflect mechanisms driving disease development or are consequences of the diseases themselves. Moreover, pharmacological treatments may have influenced the levels of certain biomarkers, representing a potential limitation of this study. Finally, the absence of longitudinal biomarker data precludes the exploration of the dynamic relationship between the changes in serum biomarkers and multimorbidity.

In conclusion, our findings—reinforced by the external validation of the longitudinal analysis—suggest that several biological processes are implicated in the development of multimorbidity, operating through either shared or specific pathophysiological mechanisms that may drive the development of diverse patterns of multimorbidity. Metabolic disturbances emerged as a central process underlying the development and accumulation of multimorbidity. If corroborated by further research, these biological processes could represent actionable targets for interventions aimed at slowing the accumulation of chronic diseases and mitigating their associated adverse outcomes.

## Methods

### Study population

This prospective observational study utilized data from SNAC-K (www.snac-k.se), involving adults aged 60 years or older, residing in a central area of Stockholm, Sweden. In the initial phase (2001–2004), participants were randomly selected and stratified into 11 age cohorts (60, 66, 72, 78, 81, 84, 87, 90, 93, 96 and over 99 years of age). A total of 3,363 individuals were included and followed every 6 years (for ages 60–72 years) or 3 years (for ages ≥78 years)^56^. The present study included data from the baseline to the fifth follow-up, covering the period from 2001 to 2019, with a maximum follow-up duration of 15 years. From the baseline cohort (*n* = 3,363), individuals with at least one missing blood-based biomarker measurement were excluded (*n* = 1,116), resulting in a final study sample of 2,247 participants. Overall, excluded participants were older, more likely to be female and more dependent on activities of daily living. They had a higher number of chronic diseases and exhibited reduced cognitive function. SNAC-K was approved by the Regional Ethical Review Authority in Stockholm (Dnrs: KI 01-114, 04-929/3, Ö26-2007, 2009/595-32, 2010/447-31/2, 2013/828-31/3, and 2016/730-31/1), and written informed consent was obtained from participants or next of kin.

To validate our findings in an independent cohort, we used data from the BLSA, a continuously-enrolled cohort study of community-dwelling adults started in 1958. Enrolled participants are followed up at age-dependent intervals: every 4 years, for individuals younger than 60 years, every 2 years for those aged 60–79 years, and every year for those aged 80 years or older. Further details on the study design are published elsewhere^57^. This study incorporated data from participants’ first assessment with complete information through a maximum follow-up duration of 15 years. Individuals aged 60 years or older with available data on baseline blood-based biomarkers, key demographic characteristics and chronic conditions were included, obtaining a final study sample of 522 participants followed up on average 6.9 years (s.d. = 4.34). This subset of BLSA participants is, on average, slightly older than other participants aged 60 years or older observed during the same period (7 August 2006 to 4 March 2025). The BLSA study was approved by the National Institutes of Health Intramural Research Program Institutional Review Board, and informed consent was obtained from each participant.

The results of this study are reported in keeping with the Strengthening the Reporting of Observational Studies in Epidemiology recommendations^58^.

### Data collection and definitions

In the SNAC-K cohort, a comprehensive data collection process, conducted by a multidisciplinary team of physicians, nurses and psychologists, was carried out during each visit at a dedicated research center. Consistent protocols were followed throughout the clinical examinations, in-person interviews and laboratory measurements to ensure data collection uniformity. Home visits were arranged for individuals who could not attend the visit site.

For the BLSA cohort, assessments were performed by the study staff either at the clinical research unit of the Intramural Research Program of the National Institute on Aging or at the individual’s home. These assessments encompassed interviews, clinical examinations and laboratory tests.

#### Disease assessment

In SNAC-K, diseases were identified at each visit through anamnestic medical history, physical examination and face-to-face and/or proxy interviews. Additional diseases were identified based on laboratory parameters, medication usage and the Swedish National Patient Register. These diagnoses were subsequently coded according to the 10th revision of the International Classification of Diseases (ICD-10). An international team of physicians and epidemiologists classified diseases as chronic when they persisted over time and were associated with either ongoing disability or the need for prolonged care, treatment or rehabilitation. The diseases identified as chronic were then grouped into 60 broad categories as previously described^3^. Incident cases of dementia, heart failure and ischemic heart disease were defined as new diagnoses made during the follow-up. A depressive episode was defined as the occurrence of either major or minor depression, as previously described^59^, during the follow-up.

In the BLSA cohort, diseases were identified based on a combination of clinical observations during comprehensive physical examination, self-reported medical history, clinical laboratory parameters and medication use. Where possible classifications were made based upon the 9th revision of the ICD (ICD-9) codes, and medications were coded according to the Anatomical Therapeutic Chemical (ATC) system.

#### Blood-based biomarkers

In the SNAC-K cohort, nonfasting venous blood samples were collected after informed consent. Blood was allowed to clot, and the serum separated from the clotted venipuncture sample was stored at −80 °C at the Karolinska Institutet BioBank. The 54 blood-based biomarkers examined at baseline were selected based on a comprehensive literature review and expert consensus. They encompass key biological processes—metabolism, inflammation, vascular function and neurodegeneration—relevant to aging and common geriatric syndromes (for example, multimorbidity, dementia and frailty), as well as routinely collected clinical parameters (for example, hemoglobin and creatinine). Several laboratory techniques were used for biomarker quantification; further details on biomarkers and assay platforms are provided in Supplementary Tables 4 and 5.

For BLSA, venous samples are collected after an overnight fast at each study visit. Clinical laboratory measurements are completed by the Clinical Laboratory Improvement Amendments certified clinical laboratory of MedStar Harbor Hospital. Samples for research measurements are prepared and stored by the Intramural Research Program of the National Institute on Aging Clinical Core Laboratory and Biorepository. Sample processing begins within 2 hours of collection with samples stored at ≤−70 °C.

#### Standard blood tests

As part of the SNAC-K protocol, standard blood tests were carried out at baseline in the hematological and biochemical laboratory at the Karolinska Hospital, in accordance with standardized institutional protocols, during the 12 hours following the assessment. The routinely performed measurements included: complete blood count, hemoglobin, total cholesterol, albumin, alkaline phosphatase, calcium, creatinine, folic acid, gamma-glutamyl transferase, thyroxine, HbA1c, thyroid stimulating hormone and vitamin B12.

In the BLSA, albumin, gamma-glutamyl transferase and HbA1c are measured as part of standard clinical laboratory panels. Fasting insulin and leptin are measured in the Intramural Research Program of the National Institute on Aging Laboratory of Clinical Investigation by enzyme-linked immunosorbent assay (ALPCO and Linco Research, Inc.). Fasting insulin is measured as part of an oral glucose tolerance test in a sample collected after an overnight fast but before glucose ingestion.

#### Protein biomarkers quantification by multiplexed immunoassay

In the SNAC-K cohort, quantification of protein biomarkers in serum was carried out at the Affinity Proteomics unit at SciLifeLab (Stockholm) using bead-based multiplexed assays performed with a Luminex system or Quanterix’s Single Molecule Arrays (Simoa). Biomarkers associated to inflammation and vascular disease were analyzed using a custom-designed magnetic Luminex assay—human premixed multi-analyte assay (Luminex Corporation). Samples were diluted following manufacturer instructions, processed in a 384 format using a validated standard operating procedure and analyzed on a FlexMap 3D (Luminex). The software xPONENT (Luminex) provides median fluorescence intensity values for samples and standards. Median fluorescence intensity is delivered for all proteins and represents a value for relative quantification. Curve fitting, extrapolation of concentrations and graphical representation were performed with Belysa Immunoassay Curve Fitting Software (Millipore), a software environment for the secondary analysis of data acquired from Luminex instruments. Standard curves were generated using a five-parameter logistic curve fit.

NfL and GFAP were assayed using the Simoa Neurology 2-Plex B kit; Aβ40, Aβ42 and T-tau were measured using the Simoa Neurology 3-Plex A kit and ptau181 was quantified using the Simoa ptau181 Advantage V2 kit. For each kit, 25 μl of sample were diluted 1:4 and the assays were performed according to manufacturer instructions. The Quanterix instrument provides average enzyme per bead values for calibrators, controls and samples. Curve fitting, extrapolation of concentrations and graphical representations are automatically performed within the Quanterix SR-X software using calibrators, a series of known concentrations of an analyte, and a four-parameter logistic curve fit. Data below the limit of detection were replaced using a not missing at random strategy, through single-value imputation, with a value of zero.

Baseline interleukin (IL-1β, IL-6, IL-8, IL-10 and IL-12p70), INFγ and TNF were measured at Accelerator Laboratory Services (Quanterix) using Simoa CorPlex Human Cytokine Panel 1 on the Quanterix SP-XTM imaging and analysis platform.

The full list of biomarkers analyzed, technology, catalog, lot number of the reagents used and analytical parameters for each assay are summarized in Supplementary Table 5. The average of intra and inter coefficient variations were calculated on a pool of samples representative of the whole SNAC-K sample set. The pool was analyzed in 8 replicates for each 384 plate (samples were distributed in 7 plates) in the Luminex assays and in three replicates in the Quanterix assays (samples were distributed in 39 plates). Average concentration in the pool and intra- and interplate coefficient variations are indicated for each protein. The average of intraplate coefficient variations is reported.

In the BLSA, GDF15 and Cystatin C were measured using the 7k SomaScan assay v4.1 (SomaLogic). Data were normalized and cross-batch harmonization was performed as previously described^60^.

Baseline biomarkers were employed as continuous variables and further converted into *z*-score to enable standardized comparisons.

### Covariates

Sociodemographic data were collected through self-reported and/or nurse-administered questionnaires during SNAC-K visits. For people with cognitive impairment, information was gathered by interviewing a proxy or caregiver. Sociodemographic variables included age, sex, education level (elementary versus high school or above), marital status (partnered versus unpartnered) and residential status (institutionalized versus noninstitutionalized). In addition, clinical and functional status were collected through physical examination, including BMI, the number of activities of daily living lost, physical activity level (active versus inactive, based on self-reported intensity and frequency of physical activity engagement^61^), global cognitive function (evaluated through the MMSE score) and the number of prescribed daily medications (self-reported or obtained from medical records for participants who were institutionalized). All-cause mortality was retrieved through the Swedish Cause of Death Register and survival status was censored at the end of the study period.

Demographic characteristics of BLSA participants are collected in structured interviews conducted by trained study staff. Anthropometric measurements, including BMI, are objectively assessed using standardized protocols.

### Statistical analyses

Baseline characteristics of the sample are reported as mean (s.d.) or median (interquartile range) for continuous variables, and count (%) for categorical variables.

#### Multimorbidity measures and latent class analysis

**Extended Data Fig. 4:**
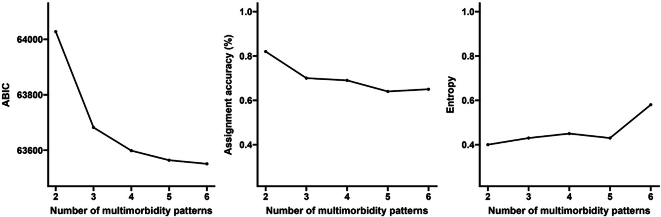
LCA metrics obtained from models with different numbers of multimorbidity patterns. ABIC of LCA models with different numbers of classes (that is, multimorbidity patterns). Both the 5-class and 6-class solutions yielded similarly low ABIC values, but the 6-class model exhibited patterns that were not clearly clinically interpretable, as well as a pattern with a very low prevalence. Assignment accuracy (that is, mean diagonal of the misclassification matrix) and entropy obtained by 5-fold cross-validation. Although entropy was higher for the 6-class solution, the assignment accuracy confirmed similar behavior of the 5- and 6-class solutions. Both indices confirmed satisfactory class separation, considering that the classes represent co-occurrence of chronic diseases. ABIC = Adjusted Bayesian Information Criterion.

Multimorbidity measures were operationalized as follows. First, multimorbidity was defined as the number of chronic conditions, both at the cross-sectional and longitudinal levels. In addition, baseline chronic conditions with a prevalence of at least 2% in the overall multimorbid population were used to identify homogeneous groups of participants with multimorbidity (that is, two or more diseases), sharing similar combinations of chronic diseases (Supplementary Table 8)^6,62^. These baseline multimorbidity patterns were identified through LCA and further validated against several distal outcomes (Supplementary Tables 2 and 3). In the context of multimorbidity patterns identified through LCA, the term ‘homogeneous’ refers to the fact that participants within the same pattern tend to exhibit similar combinations of chronic conditions. The optimal number of patterns was determined using the adjusted Bayesian Information Criterion and theoretical interpretability (Extended Data Fig. 4). In addition, LCA models with different numbers of patterns were also compared in terms of entropy and assignment accuracy through five-fold cross validation to assess the stability and separation of the latent classes (Extended Data Fig. 4). Each pattern was labeled based on the distinctive set of chronic diseases that were disproportionately represented, that is overexpressed within that group. Chronic conditions were considered to be overexpressed in each pattern if they had an O/E ratio of ≥2 and an exclusivity of ≥25% (Supplementary Table 8)^6,62–64^. The O/E ratio was calculated by dividing the prevalence of a given disease within a specific pattern by its prevalence in the overall multimorbid population, while exclusivity was calculated by dividing the absolute frequency of a given disease within a specific pattern by its frequency in the overall multimorbid population. Furthermore, the LCA model provides, for each participant, a probability of belonging to one of the latent multimorbidity patterns based on their combination of baseline chronic diseases. While the specific disease combination makes participants more likely to belong to that pattern, they may still exhibit additional chronic diseases not overexpressed in their assigned pattern. To give a descriptive overview of the five patterns in terms of key baseline characteristics, participants were assigned to the pattern with the highest probability of membership (Supplementary Table 1). However, when incorporating classes derived from a LCA model into subsequent statistical models, it is essential to account for the inherently probabilistic nature of class membership. Assigning participants solely to their most likely class can lead to overly confident and potentially biased associations in downstream models. To account for the probabilistic nature of pattern membership, each subsequent analysis concerning multimorbidity patterns was performed across 1,000 resampled multimorbidity pattern membership datasets derived from the LCA models. Specifically, for each participant, class membership was sampled 1,000 times from a multinomial distribution parameterized by their class membership probabilities. Subsequently, each dataset was used to fit a regression model, enabling participants to contribute to each multimorbidity pattern in proportion to their membership probabilities across iterations.

For the clinical validation of the multimorbidity patterns against distal outcomes, the following models were employed across the 1,000 LCA-resampled multimorbidity pattern membership datasets: logistic regression models adjusted for age, sex and education for incident cases of dementia, heart failure and ischemic heart disease; Andersen–Gill Cox model for recurrent depressive events with age as time scale, adjusted by sex, education and past depressive episodes (time-dependent covariate) and Cox model with age as time scale, adjusted for sex and education for all-cause mortality. All models were fitted using, as the independent variable, the six baseline multimorbidity groups using no multimorbidity as the reference group. For each analysis, the final coefficients with their relative standard errors were calculated using Rubin’s rule.

#### Cross-sectional analysis

To assess the relationship between baseline biomarkers, a correlation matrix was constructed using Spearman’s rank correlation coefficients. To account for potential confounding effects of age, age-adjusted correlations were also calculated (Extended Data Fig. 1).

To address multicollinearity and select the most important predictors, LASSO with L1 regularization was performed to model the associations between baseline biomarkers and multimorbidity measures. A random seed was set for reproducibility. For the association between biomarkers and the baseline number of diseases, Gaussian LASSO adjusted for age, sex and education was applied with cross validation using 200 folds (10% of the sample) to optimize the regularization parameter. Model selection was based on mean squared error, and lambda 1se criterion was chosen to select the most regularized model such that the cross-validated error was within one standard error of the minimum.

Similarly, multinomial LASSO adjusted for age, sex and education was used to assess associations between biomarkers and baseline multimorbidity patterns, with a 200-fold cross validation. The multinomial LASSO was performed across 1,000 resampled multimorbidity pattern membership datasets derived from the LCA models. For each biomarker, the final coefficient was calculated as the mean of the nonzero coefficients across models. Model selection was based on deviance, and lambda 1se was applied as regularization criterion. Coefficients were reported using the No-multimorbidity group as the reference category. Biomarkers were considered repeatedly associated with a given pattern if they were selected in at least 70% of the LASSO models. Final coefficients for these biomarkers were calculated as the mean of the nonzero coefficients across the models.

The nonzero coefficients of the LASSO models indicate that the corresponding biomarkers contribute independently to the prediction of the dependent variables (that is, the multimorbidity measures). The absolute value of the coefficient reflects the strength of the association, while the sign of the coefficient indicates the effect (direct or reverse). Coefficients that are shrunk to zero imply that the corresponding biomarkers do not contribute meaningfully to the model when considering all other included variables.

#### Longitudinal analysis

Linear mixed-effect models with random intercepts and random slopes were used to assess the accumulation of diseases over time. The model was adjusted for age, sex and education. The estimated random slopes from the linear mixed-effect model were then used as outcomes in the Gaussian LASSO model to identify the biomarkers associated with a steeper trajectory of accumulation of disease.

#### Principal component analysis

To identify clusters and reduce dimensionality, PCA was performed. For each multimorbidity measure, PCA was performed on biomarkers selected by the LASSO analysis (that is, those with nonzero coefficients in the LASSO models), both in cross-section and longitudinally. The number of the principal components considered was selected considering the percentage of the total explained variance (minimum threshold set at 40%).

#### External validation of longitudinal findings

We first harmonized the chronic conditions available in BLSA using ICD and ATC codes, as well as study visit data to align with those in the SNAC-K dataset. The baseline characteristics of the BLSA study sample are reported in Supplementary Table 7. Due to limited biomarker availability in the BLSA, it was not feasible to replicate the full LASSO models developed in SNAC-K cohort. Instead, we validated the results of the LASSO analysis—which identified biomarkers associated with the rate of disease accumulation in SNAC-K, our most important results—by assessing the predictive accuracy of the LASSO-derived model in an independent cohort. We used MSE as the accuracy metric, consistent with the metric used during model training. This approach represents a standard and widely-accepted method for validating LASSO results, as it tests the model’s ability to generalize by applying it to an external dataset and comparing predictive performance^65,66^. This validation approach was particularly appropriate given that the BLSA cohort represents approximately 25% of the size of SNAC-K cohort and that not all biomarkers were measured, making full model retraining unfeasible but still allowing for a meaningful assessment of generalizability through external prediction. To evaluate the external performance of the SNAC-K LASSO-selected biomarkers in the BLSA dataset, we applied a multistep approach: (1) individual rates of disease accumulation in BLSA were estimated using a linear mixed-effects model with random intercept and random slope; (2) an age-adjusted linear regression model was fitted in BLSA using the estimated slopes as outcomes and the selected biomarkers as predictors; (3) the SNAC-K LASSO-derived coefficients were then applied to the BLSA data to generate predicted individual rates of disease accumulation; and (4) predictive accuracy was assessed by calculating the MSE between the predicted and observed slopes, and compared to the MSE obtained in SNAC-K. Statistical analysis was conducted using R software (version 4.2.3). Specifically, poLCA, glmnet, lme4, factoextra, corrplot, survival and ggplot2 packages were used for the analysis. The level of statistical significance was set at *P* < 0.05.

### Reporting summary

Further information on research design is available in the Nature Portfolio Reporting Summary linked to this article.

## Online content

Any methods, additional references, Nature Portfolio reporting summaries, source data, extended data, supplementary information, acknowledgements, peer review information; details of author contributions and competing interests; and statements of data and code availability are available at 10.1038/s41591-025-04038-2.

## Supplementary information

Supplementary Information Supplementary Tables 1–8.

Reporting Summary

## Data Availability

SNAC-K data are sensitive; thus, they cannot be shared publicly but raw and analyzed deidentified data can be requested by qualified researchers at https://www.snac-k.se/. The request will be reviewed to ensure confidentiality obligations and intellectual property. A datasharing agreement must be signed before data release. BLSA data are available to qualified researchers through submission of proposals at https://www.blsa.nih.gov/.

## Code availability

No custom code or mathematical algorithm was employed in the study. Analysis scripts for this study are available at https://github.com/AliRita/Biomarkers-and-Multimorbidity-study.
