# Supplementary material for: Shared and specific blood biomarkers for multimorbidity
Source: Nat Med. 2026 Jan 2;32(2):736–45. doi: 10.1038/s41591-025-04038-2 (PMC12920107; doi:10.1038/s41591-025-04038-2)
Supplement: Supplementary file 1 — Supplementary Tables 1–8. [file 41591_2025_4038_MOESM1_ESM.pdf]

nature medicine

Supplementary information https://doi.org/10.1038/s41591-025-04038-2

Shared and specific blood biomarkers for multimorbidity

In the format provided by the authors and unedited

Shared and specific blood biomarkers for multimorbidity

Supplementary Tables

Supplementary Table 1. Baseline sample characteristics overall and by patterns of multimorbidity

Supplementary Table 2. Association between baseline multimorbidity patterns and distal outcomes

Supplementary Table 3. Association between baseline multimorbidity patterns and all-cause mortality

Supplementary Table 4. Baseline blood-based biomarkers overall and by patterns of multimorbidity

Supplementary Table 5. Biomarkers' names, acronyms, and analytical platform

Supplementary Table 6. Loadings of each LASSO-selected biomarker to the first five principal components

Supplementary Table 7. Baseline BLSA sample characteristics

Supplementary Table 8. Conditional disease prevalence across multimorbidity patterns derived from LCA model with corresponding overexpression measures

Supplementary Table 1. Baseline sample characteristics overall and by patterns of multimorbidity

Baseline characteristics	**Overall 2247**	**No multimorbidity 337 (15%)**	**Unspecific 926 (41.2%)**	**Neuropsychiatric 74 (3.3%)**	**Psychiatric/Respiratory 345 (15.4%)**	**Sensory impairment/Anemia 361 (16%)**	**Cardiometabolic 204 (9.1%)**
Age, mean (SD)	72.71 (10.7)	64.68 (6.5)	69.86 (8.7)	86.95 (8.8)	70.59 (8.9)	81.07 (9.2)	82.51 (9.1)
Women, n (%)	1383 (61.5)	179 (53.1)	526 (56.8)	54 (73.0)	265 (76.8)	240 (66.5)	119 (58.3)
BMI, mean (SD)	25.66 (4.1)	24.71 (2.8)	26.67 (4.2)	21.77 (3.7)	25.45 (3.8)	24.56 (3.6)	25.69 (5.2)
Education: High school or above, n (%)	1894 (84.3)	314 (93.2)	806 (87.0)	47 (63.5)	287 (83.2)	278 (77.0)	162 (79.4)
Partnered, n (%)	1091 (48.6)	223 (66.2)	517 (55.9)	18 (24.3)	143 (41.7)	120 (33.2)	70 (34.3)
Institutionalized, n (%)	37 (1.6)	-	3 (0.3)	13 (17.6)	2 (0.6)	13 (3.6)	6 (2.9)
Clinical and functional status							
1+ ADL lost, n (%)	96 (4.3)	1 (0.3)	11 (1.2)	32 (43.2)	10 (2.9)	19 (5.3)	23 (11.3)
MMSE score, mean (SD)	28.29 (2.9)	29.13 (1.9)	28.93 (1.6)	19.88 (6.7)	28.89 (1.4)	27.59 (2.9)	27.30 (3.3)
Active physical level, n (%)	1589 (70.7)	274 (81.3)	738 (79.7)	15 (20.3)	236 (68.4)	225 (62.3)	101 (49.5)
N° of chronic diseases, mean (SD)	3.85 (2.4)	0.75 (0.4)	3.17 (1.2)	6.41 (2.1)	4.41 (1.8)	5.24 (1.8)	7.76 (2.2)
N° of drugs, mean (SD)	3.67 (3.3)	1.18 (1.6)	2.72 (2.5)	6.11 (3.9)	4.89 (3.4)	4.45 (2.9)	7.74 (3.5)

SD = standard deviation; ADL = activity of daily living; BMI = body mass index; MMSE = mini-mental state examination; eGFR = estimated glomerular filtration rate.
The mismatch between total numbers and sample sizes is due to missing data. Missing values for civil status = 2 for psychiatric/respiratory; 1 for unspecific; BMI = 1 for no-multimorbidity; 17 for unspecific; 29 for neuropsychiatric; 6 for psychiatric/respiratory; 25 for sensory impairment/anemia; 18 for cardiometabolic; MMSE = 2 for no-multimorbidity; 2 for unspecific; 2 for psychiatric/respiratory; 1for neuropsychiatric; 1 for cardiometabolic.

Supplementary Table 2: Association between baseline multimorbidity patterns and distal outcomes

	Recurrent depressive episodes (N = 2247)	Incident dementia (N = 1621)	Incident heart failure (N = 1561)	Incident ischemic heart disease (N = 1462)
**Multimorbidity groups**	**HR (95%CI)**	**OR (95%CI)**	**OR (95%CI)**	**OR (95%CI)**
No-multimorbidity	Ref.	Ref.	Ref.	Ref.
Unspecific pattern	1.29 (0.70-2.37)	0.98 (0.93-1.03)	1.00 (0.94-1.05)	1.08 (0.99-1.17)
Neuropsychiatric pattern	4.08 (1.55-10.76)	1.81 (1.63-2.01)	1.22 (1.11-1.34)	1.14 (1.01-1.30)
Psychiatric/Respiratory pattern	2.42 (1.32-4.43)	0.97 (0.92-1.03)	0.99 (0.93-1.04)	1.06 (0.97-1.15)
Sensory impairment/Anemia pattern	1.59 (0.81-3.09)	1.01 (0.96-1.07)	0.98 (0.93-1.04)	1.09 (1.00-1.19)
Cardiometabolic pattern	1.98 (0.94-4.19)	1.03 (0.97-1.09)	1.84 (1.71-1.98)	1.65 (1.50-1.83)

Recurrent depressive episodes: Andersen-Gill Cox model for recurrent events with age as time scale, adjusted by sex, education, and past depressive episodes (time-dependent).
Incident dementia: logistic regression model adjusted by age, sex, and education.
Incident heart failure: logistic regression model adjusted by age, sex, and education.
Incident ischemic heart disease: logistic regression model adjusted by age, sex, and education.
Note: Logistic regression models were fitted for each outcome, excluding prevalent cases at baseline.
CI = confidential interval; HR = hazard ratio; OR = odds ratio.

Supplementary Table 3: Association between baseline multimorbidity patterns and all-cause mortality

	All-cause mortality (n = 2247)
**Multimorbidity groups**	**HR (95%CI)**
No-multimorbidity	Ref.
Unspecific pattern	1.53 (1.14-2.06)
Neuropsychiatric pattern	2.85 (1.92-4.24)
Psychiatric/Respiratory pattern	1.85 (1.34-2.54)
Sensory impairment/Anemia pattern	1.69 (1.24-2.31)
Cardiometabolic pattern	2.56 (1.86-3.52)

Cox model with age as time scale, adjusted by sex and education.
CI = confidential interval; HR = hazard ratio.

Supplementary Table 4. Baseline serum biomarkers overall and by patterns of multimorbidity

Serum biomarkers	Overall<br>2247	No multimorbidity<br>337 (15%)	Unspecific<br>926 (41.2%)	Neuropsychiatric<br>74 (3.3%)	Psychiatric/Respiratory<br>345 (15.4%)	Sensory impairment/Anemia<br>361 (16%)	Cardiometabolic<br>204 (9.1%)
Metabolism							
Adiponectin (µg/mL), median [IQR]	15.00<br>[11.50, 19.30]	14.46<br>[11.10, 18.58]	13.80<br>[10.70, 17.53]	19.90<br>[14.99, 24.59]	15.22<br>[12.10, 19.40]	16.61<br>[12.94, 21.06]	16.37<br>[12.53, 21.83]
Albumin (g/L), median [IQR]	42.00<br>[40.00, 44.00]	42.00<br>[40.00, 44.00]	42.00<br>[40.00, 44.00]	39.00<br>[37.00, 41.00]	41.00<br>[39.00, 44.00]	41.00<br>[38.00, 43.00]	41.00<br>[39.00, 43.00]
Alkaline phosphatase (ukat/L), median [IQR]	2.50<br>[2.10, 3.00]	2.40<br>[2.00, 2.80]	2.50<br>[2.10, 3.00]	2.80<br>[2.32, 3.48]	2.50<br>[2.10, 3.00]	2.60<br>[2.10, 3.20]	2.60<br>[2.18, 3.32]
Calcium (mmol/L), median [IQR]	2.45<br>[2.39, 2.52]	2.45<br>[2.39, 2.50]	2.46<br>[2.41, 2.52]	2.40<br>[2.34, 2.45]	2.45<br>[2.39, 2.52]	2.43<br>[2.36, 2.51]	2.45<br>[2.39, 2.52]
Cholesterol (mmol/L), median [IQR]	6.00<br>[5.30, 6.70]	5.70<br>[5.20, 6.10]	6.30<br>[5.60, 6.90]	5.35<br>[4.82, 5.80]	6.00<br>[5.40, 6.90]	5.80<br>[5.20, 6.70]	5.50<br>[4.60, 6.40]
C-Peptide (ng/mL), median [IQR]	2.03<br>[1.27, 2.99]	1.68<br>[1.13, 2.38]	2.06<br>[1.29, 2.97]	2.42<br>[1.65, 3.72]	1.78<br>[1.15, 2.86]	2.12<br>[1.40, 3.01]	2.71<br>[1.75, 3.89]
Creatinine (µmol/L), median [IQR]	86.00<br>[77.00, 96.00]	82.00<br>[76.00, 89.00]	85.00<br>[77.00, 94.00]	91.00<br>[73.25, 103.00]	81.00<br>[74.00, 91.00]	90.00<br>[81.00, 104.00]	103.00<br>[89.75, 123.00]
Cystatin C (mg/L), median [IQR]	0.73<br>[0.63, 0.88]	0.66<br>[0.58, 0.73]	0.70<br>[0.61, 0.80]	0.90<br>[0.80, 1.16]	0.71<br>[0.63, 0.83]	0.85<br>[0.74, 1.04]	0.99<br>[0.80, 1.25]
Folic acid (nmol/L), median [IQR]	232.00<br>[183.00, 308.00]	232.00<br>[194.00, 284.00]	235.00<br>[189.00, 305.00]	185.00<br>[134.75, 303.50]	235.00<br>[177.00, 322.00]	222.00<br>[171.00, 322.00]	234.00<br>[182.00, 346.50]
GDF-15 (ng/mL), median [IQR]	0.92<br>[0.70, 1.29]	0.73<br>[0.59, 0.96]	0.86<br>[0.67, 1.10]	1.56<br>[1.11, 1.95]	0.89<br>[0.72, 1.17]	1.24<br>[0.90, 1.59]	1.51<br>[1.12, 2.05]
γ-GT (ukat/L), median [IQR]	0.42<br>[0.29, 0.67]	0.37<br>[0.28, 0.55]	0.47<br>[0.32, 0.73]	0.35<br>[0.23, 0.58]	0.39<br>[0.27, 0.60]	0.35<br>[0.26, 0.54]	0.52<br>[0.34, 0.96]
Hemoglobin (g/L), median [IQR]	138.00<br>[130.00, 146.00]	140.00<br>[133.00, 148.00]	141.00<br>[134.00, 149.00]	128.50<br>[120.25, 138.75]	136.00<br>[130.00, 142.00]	131.00<br>[123.00, 141.00]	134.00<br>[122.00, 142.00]
HbA1C (%), median [IQR]	4.50<br>[4.20, 4.70]	4.40<br>[4.10, 4.60]	4.50<br>[4.20, 4.70]	4.50<br>[4.20, 4.77]	4.40<br>[4.20, 4.70]	4.50<br>[4.30, 4.80]	4.70<br>[4.40, 5.40]
IGFBP1 (ng/mL), median [IQR]	17.92<br>[11.76, 32.85]	16.21<br>[10.95, 26.16]	15.31<br>[10.70, 25.21]	41.16<br>[21.18, 76.04]	17.75<br>[12.01, 32.51]	25.95<br>[15.76, 50.33]	21.90<br>[13.46, 47.66]
Insulin (pmol/L), median [IQR]	168.88<br>[114.28, 264.75]	146.60<br>[104.50, 210.28]	178.84<br>[119.63, 275.62]	187.94<br>[117.53, 302.26]	151.58<br>[107.78, 258.08]	162.18<br>[107.78, 246.97]	206.42<br>[145.01, 341.71]
Leptin (ng/mL), median [IQR]	15.55<br>[9.20, 25.67]	12.39<br>[7.46, 19.76]	16.69<br>[10.17, 26.32]	9.60<br>[6.97, 18.27]	17.26<br>[10.90, 27.92]	14.77<br>[8.47, 26.47]	18.27<br>[9.72, 31.61]
T4 (pg/mL), median [IQR]	15.00<br>[14.00, 17.00]	15.00<br>[14.00, 16.00]	15.00<br>[14.00, 16.00]	15.00<br>[14.00, 17.00]	15.00<br>[14.00, 17.00]	15.00<br>[14.00, 16.00]	15.00<br>[14.00, 17.00]
TSH (mIU/L), median [IQR]	1.50<br>[0.99, 2.10]	1.50<br>[1.10, 2.00]	1.50<br>[1.10, 2.10]	1.30<br>[0.73, 1.87]	1.40<br>[0.77, 2.00]	1.40<br>[1.00, 2.20]	1.60<br>[1.00, 2.50]
Vitamin B12 (pg/mL), median [IQR]	348.00<br>[279.00, 469.00]	335.00<br>[277.00, 414.00]	341.00<br>[276.00, 458.00]	422.00<br>[290.25, 745.75]	343.00<br>[284.00, 479.00]	350.00<br>[271.00, 500.00]	390.50<br>[295.25, 525.00]
Inflammation							

β2-Microglobulin (µg/mL), median [IQR]	2.21 [1.74, 2.92]	1.87 [1.56, 2.31]	2.05 [1.66, 2.53]	3.73 [2.56, 4.89]	2.13 [1.68, 2.78]	2.77 [2.08, 3.66]	3.47 [2.51, 4.88]
CRP (mg/L), median [IQR]	1.63 [0.74, 3.48]	1.13 [0.51, 2.47]	1.56 [0.74, 3.17]	1.72 [0.61, 4.95]	1.93 [0.87, 4.69]	1.50 [0.74, 3.53]	2.59 [1.31, 5.63]
CCL2 (pg/mL), median [IQR]	354.17 [278.99, 446.68]	351.73 [270.52, 431.86]	363.15 [290.44, 454.20]	346.78 [254.67, 420.50]	353.80 [264.09, 460.29]	347.23 [278.15, 440.48]	336.76 [282.59, 426.78]
CCL3 (pg/mL), median [IQR]	1126.69 [1002.20, 1251.26]	1118.30 [999.63, 1235.93]	1133.83 [1007.75, 1253.32]	1152.74 [1039.81, 1290.71]	1117.22 [993.62, 1258.70]	1105.75 [981.04, 1230.64]	1124.40 [1007.56, 1271.57]
CCL4 (ng/mL), median [IQR]	1.88 [1.64, 2.17]	1.86 [1.62, 2.14]	1.90 [1.65, 2.17]	1.90 [1.77, 2.21]	1.86 [1.64, 2.18]	1.87 [1.61, 2.10]	1.88 [1.67, 2.21]
CCL11(pg/mL), median [IQR]]	359.30 [306.19, 415.78]	353.21 [305.37, 405.07]	358.79 [306.68, 415.80]	381.06 [341.40, 433.52]	354.57 [307.03, 408.64]	359.30 [298.88, 418.47]	358.79 [300.48, 421.12]
CXCL10 (pg/mL), median [IQR]	51.17 [40.02, 67.56]	44.57 [37.18, 56.40]	49.21 [39.00, 63.15]	63.30 [48.89, 85.99]	49.94 [39.85, 67.29]	55.50 [43.77, 76.72]	65.50 [47.75, 88.20]
IFN-γ (pg/mL), median [IQR]	0.08 [0.05, 0.14]	0.07 [0.04, 0.12]	0.08 [0.05, 0.13]	0.10 [0.06, 0.17]	0.09 [0.05, 0.15]	0.09 [0.05, 0.17]	0.10 [0.07, 0.19]
IL-1β (pg/mL), median [IQR]	0.05 [0.03, 0.08]	0.04 [0.03, 0.07]	0.04 [0.03, 0.07]	0.08 [0.05, 0.12]	0.05 [0.03, 0.07]	0.05 [0.03, 0.09]	0.07 [0.04, 0.10]
IL-12p70 (pg/mL), median [IQR]	0.16 [0.09, 0.30]	0.16 [0.09, 0.30]	0.15 [0.08, 0.29]	0.19 [0.09, 0.31]	0.17 [0.09, 0.32]	0.17 [0.10, 0.29]	0.21 [0.11, 0.34]
IL-6 (pg/mL), median [IQR]	1.58 [0.92, 2.80]	1.05 [0.63, 1.79]	1.41 [0.87, 2.34]	2.79 [1.55, 5.29]	1.76 [0.99, 2.84]	1.83 [1.17, 3.07]	3.05 [1.72, 5.25]
IL-8 (pg/mL), median [IQR]	16.33 [12.07, 25.58]	15.42 [10.97, 26.06]	15.85 [12.10, 23.09]	25.29 [16.38, 41.31]	15.80 [12.11, 26.00]	16.14 [12.04, 24.94]	18.75 [14.21, 32.74]
IL-10 (pg/mL), median [IQR]	0.61 [0.43, 0.93]	0.53 [0.36, 0.85]	0.58 [0.42, 0.83]	0.89 [0.71, 1.12]	0.58 [0.42, 0.89]	0.67 [0.46, 1.05]	0.78 [0.57, 1.19]
Leukocytes (10^3/uL), median [IQR]	6.70 [5.70, 7.80]	6.20 [5.40, 7.30]	6.60 [5.70, 7.68]	6.95 [5.80, 8.20]	6.80 [5.80, 8.00]	6.70 [5.50, 7.90]	7.45 [6.07, 8.70]
MPO (ng/mL), median [IQR]	109.83 [77.19, 159.84]	102.74 [72.83, 150.66]	101.97 [74.55, 147.84]	140.44 [79.08, 192.88]	116.83 [77.85, 159.71]	116.87 [83.18, 161.50]	128.72 [91.62, 212.98]
TNF-α (pg/mL), median [IQR]	2.20 [1.67, 2.93]	1.92 [1.45, 2.51]	2.13 [1.65, 2.68]	3.10 [2.28, 3.92]	2.00 [1.59, 2.64]	2.39 [1.80, 3.23]	2.77 [2.26, 3.84]
TNFRSF1B (pg/mL), median [IQR]	3803.17 [2752.97, 5104.60]	3234.45 [2464.78, 4185.04]	3621.11 [2708.93, 4752.55]	4894.33 [3707.70, 6380.93]	3540.46 [2696.57, 4870.09]	4395.27 [3230.45, 5875.03]	4974.53 [3602.88, 6444.27]
Vascular							
EGF (pg/mL), median [IQR]	202.51 [103.19, 301.25]	205.82 [96.43, 313.73]	200.85 [97.49, 308.92]	247.32 [160.05, 311.94]	198.44 [101.02, 293.16]	193.92 [108.23, 283.18]	210.86 [118.27, 299.24]
EphA2 (ng/mL), median [IQR]	2.47 [2.17, 2.75]	2.41 [2.16, 2.67]	2.48 [2.18, 2.74]	2.61 [2.32, 2.94]	2.43 [2.14, 2.70]	2.49 [2.15, 2.81]	2.50 [2.20, 2.87]
E-selectin (ng/mL), median [IQR]	32.01 [25.11, 40.13]	31.85 [24.97, 38.13]	33.98 [27.09, 42.16]	31.28 [22.97, 39.04]	31.47 [24.88, 39.62]	28.16 [23.02, 36.37]	31.99 [24.21, 40.09]
ICAM1 (ng/mL), median [IQR]	484.87 [410.88, 578.78]	457.79 [382.93, 532.15]	486.37 [416.16, 578.84]	507.09 [425.44, 597.46]	474.12 [401.95, 560.64]	496.54 [416.68, 606.12]	526.74 [448.05, 631.85]
N-cadherin (ng/mL), median [IQR]	404.26 [324.16, 531.78]	345.47 [286.67, 410.66]	379.24 [311.71, 465.29]	640.38 [419.26, 882.26]	388.20 [316.07, 472.33]	511.95 [399.90, 687.51]	661.91 [491.55, 963.60]
P-selectin (pg/mL), median [IQR]	48502.53 [40333.08, 61037.86]	46780.92 [39359.71, 58516.91]	49348.74 [40927.88, 59805.18]	53262.92 [43298.97, 70556.18]	48304.43 [40146.59, 60058.51]	47208.61 [39540.19, 60757.26]	49826.78 [38721.87, 68374.30]

VCAM1 (ng/mL), median [IQR]	766.43 [599.07, 969.64]	703.94 [541.95, 886.18]	715.91 [578.04, 900.30]	929.01 [802.67, 1173.53]	754.56 [578.30, 962.79]	839.55 [660.90, 1056.90]	981.17 [735.37, 1208.87]
VEGF (pg/mL), median [IQR]	143.37 [106.93, 198.72]	129.55 [93.56, 170.83]	145.87 [110.68, 198.85]	138.40 [91.27, 192.06]	141.99 [107.29, 197.51]	150.33 [109.13, 203.28]	166.13 [116.58, 223.09]
Neurodegeneration							
Aβ40 (pg/mL), median [IQR]	133.39 [98.18, 165.83]	116.96 [87.20, 143.77]	127.30 [94.79, 156.34]	157.11 [127.51, 228.40]	125.62 [97.17, 159.13]	151.22 [120.38, 185.70]	168.52 [121.35, 210.59]
Aβ42 (pg/mL), median [IQR]]	7.26 [5.52, 9.07]	6.86 [5.13, 8.39]	7.02 [5.40, 8.58]	7.57 [5.54, 10.49]	7.22 [5.45, 9.14]	7.97 [6.20, 9.62]	8.48 [6.57, 10.16]
Aβ42/40, median [IQR]	0.06 [0.05, 0.07]	0.06 [0.05, 0.07]	0.06 [0.05, 0.07]	0.05 [0.04, 0.06]	0.06 [0.05, 0.07]	0.05 [0.05, 0.06]	0.05 [0.05, 0.06]
α-synuclein (ng/mL), median [IQR]	1.61 [1.38, 1.92]	1.59 [1.38, 1.83]	1.61 [1.40, 1.94]	1.66 [1.47, 1.88]	1.58 [1.36, 1.91]	1.66 [1.34, 1.95]	1.66 [1.38, 2.00]
BDNF (ng/mL), median [IQR]	23.57 [18.29, 29.57]	23.31 [18.11, 29.95]	24.43 [18.89, 30.04]	21.59 [17.85, 24.92]	23.87 [18.94, 29.66]	23.50 [18.14, 29.28]	22.45 [17.03, 28.61]
GFAP (pg/mL), median [IQR]	124.20 [81.62, 194.52]	93.97 [65.20, 128.37]	110.46 [72.44, 155.93]	235.89 [168.11, 339.31]	124.76 [83.73, 181.35]	174.02 [117.26, 277.21]	172.47 [122.89, 281.29]
NfL (pg/mL), median [IQR]	18.40 [12.68, 29.41]	13.62 [10.42, 17.64]	15.72 [11.35, 22.39]	50.42 [34.00, 64.31]	17.49 [12.84, 25.79]	30.20 [21.39, 44.40]	33.75 [23.14, 53.10]
p-tau181 (pg/mL), median [IQR]	1.19 [0.75, 1.84]	0.96 [0.65, 1.32]	1.02 [0.68, 1.58]	2.16 [1.30, 2.95]	1.05 [0.66, 1.63]	1.75 [1.22, 2.70]	1.75 [1.21, 2.64]
S100B (pg/mL), median [IQR]	1205.17 [986.58, 1448.45]	1220.70 [1032.01, 1463.73]	1209.33 [981.65, 1448.45]	1250.18 [990.41, 1455.72]	1197.61 [951.86, 1427.66]	1179.16 [981.65, 1417.58]	1226.44 [974.61, 1461.74]
t-tau (pg/mL), median [IQR]]	0.85 [0.55, 1.18]	0.77 [0.51, 1.08]	0.76 [0.50, 1.08]	1.14 [0.86, 1.52]	0.81 [0.48, 1.10]	0.99 [0.71, 1.33]	1.12 [0.75, 1.54]

Aβ 40 = amyloid beta protein 40; Aβ 42 = amyloid beta protein 42; Adipo = adiponectin; ALB = albumin; ALP = alkaline phosphatase; aSyn = alpha synuclein; BDNF = Brain-derived neurotrophic factor; B2M = beta-2-microglobulin; Ca = calcium; CCL11 = C-C motif chemokine 11; CCL2 = C-C motif chemokine 2; CCL3 = C-C motif chemokine 3; CCL4 = C-C motif chemokine 11; CPEP = C-peptide; Cr = creatinine; CRP = C-reactive protein; CXCL10 = chemokine CXC ligand 10; CysC = cystatin C; EGF = Epidermal growth factor; EphA2 = ephrin type-A receptor 2; ESel = E-selectin; FA = folic acid; GDF15 = growth differentiation factor 15; GFAP = Glial fibrillary acidic protein; GGT = gamma-glutamyl transferase; Hb = hemoglobin; HbA1c = hemoglobin A1c; ICAM1 = Intercellular Adhesion Molecule 1; IFNG = Interferon gamma; IGFBP1 = Insulin-like growth factor binding protein 1; IL-1b = interleukin 1beta; IL-10 = interleukin 10; IL-12p70 = Interleukin 12p70; IL-6 = interleukin 6; IL-8 = interleukin 8; IQR = interquartile range; LEP = leptin; MPO = Myeloperoxidase; NCAD = N-Cadherin; NfL = neurofilament light chain; PSel = P-selectin; p-tau181 = phosphorylated tau 181; S100B = S100 calcium-binding protein B; T4 = Thyroxine; TC = total cholesterol; TNFa = tumor necrosis factor alpha; TNFRSF1B = tumor necrosis factor receptor superfamily member 1B; TSH = thyroid stimulating hormone; VCAM1 = vascular cell adhesion protein 1; VEGF = vascular endothelial growth factor; VitB12 = vitamin B12; WBC = leucocytes.

Supplementary Table 5. Biomarkers' names, acronyms and analytical platform

	Acronyms	Biomarkers	Conc (pg/mL)	Intra /Inter CV(%)	Reagents	Analytical platform
1	Aβ 40	Amyloid beta protein 40	117.5	2.2/5.0	Neuro 3-plex A;101995;Lot 503659	Quanterix Simoa SR-X
2	Aβ 42	Amyloid beta protein 42	6.6	4.8/7.1	Neuro 3-plex A;101995;Lot 503659	Quanterix Simoa SR-X
3	Aβ ratio	Aβ 42/40 ratio	N/A	N/A	N/A	N/A
4	Adipo	Adiponectin	$18.6 *10^6$	9.4/13.2	RnD Systems; LXSAHM-02;Lot L146268	Luminex. FlexMap 3D
5	ALB	Albumin	N/A	N/A	N/A	N/A
6	ALP	Alkaline phosphatase	N/A	N/A	N/A	N/A
7	aSyn	Alpha synuclein	1884	3.7/6.5	RnD Systems; LXSAHM-18; Lot L146266	Luminex. FlexMap 3D
8	BDNF	Brain-derived neurotrophic factor	$26.3 *10^3$	15.1/11.1	RnD Systems; LXSAHM-04; Lot L146267	
9	B2M	β-2-microglobulin	$2.8*10^6$	7.8/13.7	RnD Systems;LXSAHM-01;Lot L146270	Luminex. FlexMap 3D
10	Ca	Calcium	N/A	N/A	N/A	N/A
11	CCL11	C-C motif chemokine 11	369	4.7/7.6	RnD Systems; LXSAHM-18; Lot L146266	Luminex FlexMap 3D
12	CCL2	C-C motif chemokine 2	$12.4 *10^3$	10.6/10.1	RnD Systems; LXSAHM-02; L146269	Luminex FlexMap 3D
13	CCL3	C-C motif chemokine 3	$11.2*10^3$	3.8/8.4	RnD Systems; LXSAHM-18; Lot L146266	Luminex FlexMap 3D
14	CCL4	C-C motif chemokine 4	$2.0 *10^3$	6.7/11.2	RnD Systems; LXSAHM-18; Lot L146266	Luminex FlexMap 3D
15	CPEP	C-peptide	$2.3 *10^3$	3.9/12.3	RnD Systems; LXSAHM-18; Lot L146266	Luminex. FlexMap 3D
16	Cr	Creatinine	N/A	N/A	N/A	N/A
17	CRP	C-reactive protein	$18.6 *10^6$	14.2/11	RnD Systems; LXSAHM-02;Lot L146268	Luminex FlexMap 3D
18	CXCL10	Chemokine CXC ligand 10	61.0	8.9/6.2	RnD Systems; LXSAHM-18; Lot L146266	Luminex FlexMap 3D
19	CysC	Cystatin C	$890.9 *10^3$	6.1/5.9	RnD Systems; LXSAHM-04; Lot L146267	Luminex FlexMap 3D
20	EGF	Epidermal growth factor	219.0	12.8/14.2	RnD Systems; LXSAHM-18; Lot L146266	Luminex FlexMap 3D
21	EphA2	Ephrin type-A receptor 2	$2.6 *10^3$	5.7/9.2	RnD Systems; LXSAHM-18; Lot L146266	Luminex FlexMap 3D
22	ESel	E-Selectin	$32.4 *10^3$	15.7/7.0	RnD Systems; LXSAHM-18; Lot L146266	Luminex FlexMap 3D
23	FA	Folic acid	N/A	N/A	N/A	N/A
24	GDF15	Growth differentiation factor 15	1157	17.4/12.3	RnD Systems; LXSAHM-18; Lot L146266	Luminex. FlexMap 3D
25	GFAP	Glial fibrillary acidic protein	194.0	13.1/21.4	Neuro 2-plex B; 103520; Lot 503409	Quanterix Simoa SR-X
26	GGT	Gamma-glutamyl transferase	N/A	N/A	N/A	N/A
27	Hb	Hemoglobin	N/A	N/A	N/A	N/A
28	HbA1c	Hemoglobin A1c	N/A	N/A	N/A	N/A
29	ICAM1	Intercellular Adhesion Molecule 1	648 *103	4.3/9.3	RnD Systems; LXSAHM-18; Lot L146266	Luminex FlexMap 3D
30	IFNG	Interferon gamma	N/A	N/A	CorPlex Human Cytokine Panel 1	Quanterix Simoa SP-X
31	IGFBP1	Insulin-like growth factor binding protein 1	$27.1 *10^3$	4.7/4.7	RnD Systems; LXSAHM-18; Lot L146266	Luminex FlexMap 3D
32	IL-1b	Interleukin 1 beta	N/A	N/A	CorPlex Human Cytokine Panel 1	Quanterix Simoa SP-X
33	IL-10	Interleukin 10	N/A	N/A	CorPlex Human Cytokine Panel 1	Quanterix Simoa SP-X
34	IL-12p70	Interleukin 12p70	N/A	N/A	CorPlex Human Cytokine Panel 1	Quanterix Simoa SP-X
35	IL-6	Interleukin 6	N/A	N/A	CorPlex Human Cytokine Panel 1	Quanterix Simoa SP-X

**36**	IL-8	Interleukin 8	N/A	N/A	CorPlex Human Cytokine Panel 1	Quanterix Simoa SP-X
**37**	Insulin	Insulin	1210	4.2/12.2	RnD Systems; LXSAHM-18; Lot L146266	Luminex. FlexMap 3D
**38**	LEP	Leptin	$20.9*10^3$	11.3/12.3	RnD Systems; LXSAHM-18; Lot L146266	Luminex. FlexMap 3D
**39**	MPO	Myeloperoxidase	$144.1 *10^3$	15.6//12.8	RnD Systems; LXSAHM-04; Lot L146267	Luminex FlexMap 3D
**40**	NCAD	N-Cadherin	$513.6*10^3$	15.3/ 5.1	RnD Systems; LXSAHM-04; Lot L146267	Luminex FlexMap 3D
**41**	NfL	Neurofilament light chain	24.1	7.6/11.6	Neuro 2-plex B; 103520; Lot 503409	Quanterix Simoa SR-X
**42**	PSel	P-selectin	$54.1*10^3$	7.1/28.8	RnD Systems; LXSAHM-18; Lot L146266	Luminex FlexMap 3D
**43**	ptau181	phosphorylated tau 181	1.4	9.2/14.4	Simoa pTau-181 Advantage V2 Kit	Quanterix Simoa SR-X
**44**	S100B	S100 calcium-binding protein B	1334	6.9/25.5	RnD Systems; LXSAHM-18; Lot L146266	Luminex FlexMap 3D
**45**	T4	Thyroxine	N/A	N/A	N/A	N/A
**46**	TC	Total cholesterol	N/A	N/A	N/A	N/A
**47**	TNFa	Tumor necrosis factor alpha	N/A	N/A	N/A	N/A
**48**	TNFRSF1B	Tumor necrosis factor receptor superfamily member 1B	5340	14.5/23	RnD Systems; LXSAHM-02; L146269	Luminex FlexMap 3D
**49**	TSH	Thyroid stimulating hormone	N/A	N/A	N/A	N/A
**50**	ttau	Total tau	0.9	10.7/11.9	Neuro 3-plex A;101995;Lot 503659	Quanterix Simoa SR-X
**51**	VCAM1	Vascular cell adhesion protein 1	$834 *10^3$	18.0/17.0	RnD Systems; LXSAHM-18; Lot L146266	Luminex FlexMap 3D
**52**	VEGF	Vascular endothelial growth factor	172	8.3/5.9	RnD Systems; LXSAHM-18; Lot L146266	Luminex FlexMap 3D
**53**	VitB12	Vitamin B 12	N/A	N/A	N/A	N/A
**54**	WBC	Leucocytes	N/A	N/A	N/A	N/A

N/A = not applicable.

Supplementary Table 6. Loadings of each LASSO-selected biomarker to the first four principal components

**Number of chronic diseases**	**PC1**	**PC2**	**PC3**	**PC4**
CPEP	0.303	0.485	0.207	-0.196
Cr	0.427	-0.142	0.378	0.130
CysC	0.484	-0.217	0.093	-0.002
GDF15	0.449	-0.136	-0.030	-0.006
Hb	-0.187	0.278	0.641	0.376
HbA1c	0.155	0.261	-0.489	0.799
Insulin	0.216	0.597	0.075	-0.077
LEP	0.175	0.340	-0.385	-0.386
NfL	0.392	-0.248	-0.021	0.098
**Unspecific pattern**	**PC1**	**PC2**	**PC3**	**PC4**
Aβ42/40	-0.052	-0.072	0.283	0.048
Adipo	-0.150	0.376	-0.052	-0.371
CPEP	0.352	-0.250	-0.338	-0.038
Cr	0.268	0.248	0.064	0.526
CysC	0.264	0.420	-0.021	0.274
ESel	0.355	-0.202	0.293	-0.235
FA	0.075	-0.043	-0.018	-0.022
GDF15	0.402	0.303	0.033	-0.187
GGT	0.190	-0.113	0.321	-0.127
Hb	0.229	-0.225	0.407	0.305
HbA1c	0.190	-0.127	-0.280	0.067
ICAM1	0.175	0.061	0.335	-0.003
Insulin	0.354	-0.316	-0.285	-0.107
LEP	0.188	-0.053	-0.208	-0.222
NfL	0.150	0.406	-0.185	0.069
TC	-0.125	0.004	0.259	-0.219
VCAM1	0.249	0.260	0.160	-0.436
**Neuropsychiatric pattern**	**PC1**	**PC2**	**PC3**	**PC4**
Aβ42/40	-0.117	-0.062	0.485	0.299
ALB	-0.271	-0.152	-0.146	-0.028
CPEP	0.128	-0.485	0.081	0.014
Cr	0.403	-0.151	-0.171	0.088
CysC	0.424	-0.087	-0.113	0.156
FA	0.024	0.118	0.356	0.214
GDF15	0.366	0.002	0.194	-0.238
Hb	-0.257	-0.143	-0.050	-0.531
HbA1c	0.075	-0.350	0.105	-0.335
IGFBP1	0.299	0.260	0.008	-0.270
IL8	-0.058	-0.184	-0.642	0.070
Insulin	0.031	-0.524	0.065	0.050
LEP	-0.083	-0.402	0.265	0.001
NfL	0.447	-0.064	-0.070	0.200
TC	-0.218	-0.093	-0.159	0.510
**Psychiatric/Respiratory pattern**	**PC1**	**PC2**	**PC3**	**PC4**
Aβ42/40	0.112	0.349	-0.428	-0.082
CPEP	-0.468	0.257	0.252	0.143
Cr	-0.395	-0.228	-0.393	-0.008
CysC	-0.408	-0.452	-0.124	-0.157
FA	-0.051	-0.148	-0.174	0.475
GDF15	-0.329	-0.303	-0.138	-0.105
GGT	-0.076	0.023	-0.018	0.566
Hb	-0.085	0.163	-0.472	0.354
HbA1c	-0.251	0.041	0.183	-0.192
Insulin	-0.397	0.455	0.202	0.101

LEP	-0.311	0.243	0.066	-0.212
T4	0.065	-0.347	0.305	0.096
TC	0.023	0.164	-0.372	-0.406
**Sensory impairment/Anemia pattern**	**PC1**	**PC2**	**PC3**	**PC4**
Aβ42/40	-0.100	0.063	0.405	-0.085
CPEP	0.367	0.446	-0.124	0.056
Cr	0.501	-0.074	-0.090	0.125
CysC	0.517	-0.214	0.028	0.099
FA	0.014	0.065	-0.432	-0.414
GDF15	0.431	-0.195	0.300	-0.021
Hb	-0.136	0.302	-0.210	0.450
HbA1c	-0.002	0.105	0.449	-0.593
Insulin	0.183	0.593	0.016	-0.160
LEP	0.171	0.425	0.204	0.084
ptau181	0.243	-0.260	-0.082	-0.022
TC	-0.106	0.030	0.490	0.452
**Cardiometabolic pattern**	**PC1**	**PC2**	**PC3**	**PC4**
Aβ42/40	-0.064	-0.003	0.175	0.460
CPEP	0.176	0.399	-0.411	-0.226
Cr	0.407	-0.082	-0.123	0.121
CysC	0.474	-0.104	-0.105	0.160
FA	-0.068	-0.063	-0.529	0.018
GDF15	0.443	-0.018	0.075	-0.189
GGT	0.036	0.330	0.250	-0.290
Hb	-0.268	0.154	-0.001	-0.050
HbA1c	-0.015	0.455	0.124	0.265
Insulin	0.081	0.560	-0.228	0.017
LEP	0.208	0.348	0.183	0.351
NCAD	0.429	-0.188	-0.104	0.115
TC	0.164	0.053	0.414	0.145
VCAM1	0.199	0.036	0.307	-0.587
VitB12	0.015	-0.069	0.222	-0.029
**Rate of disease accumulation**	**PC1**	**PC2**	**PC3**	**PC4**
ALB	-0.307	0.287	-0.400	0.467
CysC	0.535	-0.394	-0.078	0.151
GDF15	0.553	-0.282	-0.256	0.087
GGT	0.135	0.274	-0.817	-0.187
HbA1c	0.293	0.415	0.129	-0.604
Insulin	0.311	0.544	0.177	0.056
LEP	0.338	0.374	0.230	0.591

Aβ 40 = amyloid beta protein 40; Aβ 42 = amyloid beta protein 42; Adipo = adiponectin; ALB = albumin; CPEP = C-peptide; Cr = creatinine; CysC = cystatin C; ESel = E-selectin; FA = folic acid; GDF15 = growth differentiation factor 15; GGT = gamma-glutamyl transferase; Hb = hemoglobin; HbA1c = hemoglobin A1c; ICAM1 = Intercellular Adhesion Molecule 1; IGFBP1 = Insulin-like growth factor binding protein 1; IL-8 = interleukin 8; LEP = leptin; NCAD = N-Cadherin; NfL = neurofilament light chain; p-tau181 = phosphorylated tau 181; T4 = Thyroxine; TC = total cholesterol; VCAM1 = vascular cell adhesion protein 1; VitB12 = vitamin B12.

Supplementary Table 7. **Baseline BLSA sample characteristics**

Baseline characteristics	Overall (n = 522)
Age, mean (SD)	75.7 (8.7)
Women, n (%)	270 (51.7)
BMI, mean (SD)	27.1 (4.7)
Education: High school or above, n (%)	518 (99.2)
N° of chronic diseases, mean (SD)	5.2 (2.7)
**Chronic conditions**	
Allergy, n (%)	-
Anemia, n (%)	94 (18)
Asthma, n (%)	36 (6.9)
Atrial fibrillation, n (%)	15 (2.9)
Autoimmune diseases, n (%)	3 (0.6)
Blindness/visual loss, n (%)	7 (1.3)
Blood/blood forming organ diseases, n (%)	7 (1.3)
Bradycardias/conduction diseases, n (%)	28 (5.4)
COPD/emphysema/chronic bronchitis, n (%)	139 (27)
Cardiac valve diseases, n (%)	-
Cataract/lens diseases, n (%)	93 (18)
Cerebrovascular diseases, n (%)	16 (3.1)
Chromosomal abnormalities, n (%)	-
Chronic infectious disease, n (%)	24 (4.6)
Chronic kidney diseases, n (%)	88 (17)
Chronic liver diseases, n (%)	3 (0.6)
Chronic pancreas/biliary tract/gallbladder diseases, n (%)	5 (1.0)
Chronic ulcer of the skin, n (%)	-
Colitis related diseases, n (%)	42 (8.0)
Deafness/hearing loss, n (%)	112 (21)
Dementia, n (%)	15 (2.9)
Depression/mood disorders, n (%)	60 (11)
Diabetes, n (%)	52 (10.0)
Dorsopathies, n (%)	47 (9.0)
Dyslipidemia, n (%)	343 (66)
Ear/nose/throat diseases, n (%)	76 (15)
Epilepsy, n (%)	1 (0.2)
Esophagus/stomach/duodenum diseases, n (%)	1 (0.2)
Glaucoma, n (%)	52 (10.0)
Heart failure, n (%)	6 (1.1)
Hematological neoplasms, n (%)	6 (1.1)
Hypertension, n (%)	253 (48)
Inflammatory arthropathies, n (%)	47 (9.0)
Inflammatory bowel diseases, n (%)	21 (4.0)
Ischemic heart diseases, n (%)	30 (5.7)
Migraine/facial pain syndrome, n (%)	1 (0.2)
Multiple sclerosis, n (%)	-
Neurotic stress/somatic diseases, n (%)	-
Obesity, n (%)	122 (23)
Osteoarthrosis/degenerative joint diseases, n (%)	297 (57)
Osteoporosis, n (%)	141 (27)
Other MSK joint diseases, n (%)	-
Other cardiovascular diseases, n (%)	-
Other digestive diseases, n (%)	-
Other eye diseases, n (%)	-

Other genitourinary diseases, n (%)	-
Other metabolic diseases, n (%)	1 (0.2)
Other neurological diseases, n (%)	-
Other psychiatric/behavioral diseases, n (%)	-
Other respiratory diseases, n (%)	-
Other skin diseases, n (%)	-
Parkinson or parkinsonism, n (%)	7 (1.3)
Peripheral neuropathy, n (%)	64 (12)
Peripheral vascular diseases, n (%)	25 (4.8)
Prostate diseases, n (%)	69 (13)
Schizophrenia/delusional diseases, n (%)	1 (0.2)
Sleep disorders, n (%)	33 (6.3)
Solid neoplasms, n (%)	51 (9.8)
Thyroid diseases, n (%)	74 (14)
Venous/lymphatic diseases, n (%)	130 (25)
**Blood biomarkers**	
Albumin (g/dL),median [IQR]	3.8 (0.5)
Cystatin C (RFU),median [IQR]	4104 (997)
GDF15 (RFU),median [IQR]	33016 (17677)
γ-GT (u/L), median [IQR]	24 (15)
HbA1c (%), median [IQR]	5.8 (0.5)
Insulin (uU/mL),median [IQR]	6.7 (6.3)
Leptin (ng/mL), median [IQR]	12.7 (19.7)

BMI = body mass index; COPD = chronic obstructive pulmonary disease; IQR = interquartile range; GDF15 = growth differentiation factor 15; γ-GT = gamma-glutamyl transferase; MSK = muscle skeletal.; RFU = relative fluorescence units; SD = standard deviation. The mismatch between total numbers and sample sizes is due to missing data. Missing values for body mass index = 1.

Supplementary Table 8. Conditional disease prevalence across multimorbidity patterns derived from LCA model with corresponding overexpression measures

Multimorbidity pattern	Disease	Prevalence (%)	O/E	Exclusivity
Unspecific	Hypertension	87	1.18	51
	Dyslipidemia	66	1.32	58
	Chronic kidney diseases	23	0.61	27
	Obesity	19	1.49	65
	Osteoarthrosis/degenerative joint diseases	13	0.88	38
	Ischemic heart diseases	12	0.67	29
	Diabetes	9	0.95	41
	Solid neoplasms	8	0.78	34
	Thyroid diseases	7	0.62	27
	Other MSK and joint diseases	5	0.71	31
	Prostate diseases	5	1.04	45
	Atrial fibrillation	4	0.41	18
	Cerebrovascular diseases	4	0.44	19
	Deafness/hearing loss	4	0.28	12
	Asthma	3	0.47	20
	Autoimmune diseases	3	0.60	26
	Colitis and related diseases	3	0.17	8
	Dorsopathies	3	0.43	19
	Esophagus/stomach/duodenum diseases	3	0.63	28
	Other genitourinary diseases	3	0.96	42
	Anemia	2	0.17	7
	COPD/emphysema/chronic bronchitis	2	0.32	14
	Depression/mood disorders	2	0.18	8
	Inflammatory arthropathies	2	0.38	16
	Migraine/facial pain syndrome	2	0.82	36
	Sleep disorders	2	0.92	40
	Cardiac valve diseases	1	0.36	16
	Cataract/lens diseases	1	0.10	4
	Dementia	1	0.08	3
	Glaucoma	1	0.17	7
	Heart failure	1	0.08	3
	Other cardiovascular diseases	1	0.20	9
	Other neurological diseases	1	0.61	27
	Other psychiatric/behavioral diseases	1	0.43	19
	Blindness/visual loss	0	0.00	0
	Bradycardias conduction diseases	0	0.22	10
	Neurotic/stress related/somatoform diseases	0	0.00	0
	Osteoporosis	0	0.00	0
	Other eye diseases	0	0.01	1
Neuropsychiatric	**Dementia**	**88**	**8.09**	**75**
	**Colitis and related diseases**	**47**	**3.26**	**30**
	Hypertension	40	0.55	5
	**Deafness/hearing loss**	**37**	**2.81**	**26**
	Heart failure	29	2.43	22
	**Cerebrovascular diseases**	**27**	**3.07**	**28**

	Chronic kidney diseases	27	0.71	7
	Anemia	24	1.76	16
	Ischemic heart diseases	24	1.40	13
	Atrial fibrillation	22	1.98	18
	Depression/mood disorders	18	1.74	16
	Thyroid diseases	17	1.44	13
	**Blindness/visual loss**	**14**	**2.79**	**26**
	Other MSK and joint diseases	14	1.94	18
	Osteoporosis	13	1.74	16
	**Other psychiatric/behavioral diseases**	**13**	**5.28**	**49**
	COPD/emphysema/chronic bronchitis	8	1.44	13
	Glaucoma	7	1.10	10
	Neurotic/stress related/somatoform diseases	7	1.98	18
	Other cardiovascular diseases	7	1.70	16
	Autoimmune diseases	6	1.16	11
	Diabetes	6	0.56	5
	Asthma	5	0.70	6
	Dorsopathies	5	0.66	6
	Dyslipidemia	5	0.10	1
	Osteoarthrosis/degenerative joint diseases	5	0.36	3
	Esophagus/stomach/duodenum diseases	4	0.90	8
	Migraine/facial pain syndrome	4	1.71	16
	Other neurological diseases	4	1.70	16
	Solid neoplasms	4	0.37	3
	Inflammatory arthropathies	3	0.57	5
	Bradycardias/conduction diseases	2	1.05	10
	Cataract/lens diseases	2	0.30	3
	Other eye diseases	2	0.33	3
	Other genitourinary diseases	1	0.39	4
	Sleep disorders	1	0.62	6
	Cardiac valve diseases	0	0.13	1
	Obesity	0	0.03	0
	Prostate diseases	0	0.08	1
**Psychiatric/Respiratory**	Hypertension	55	0.74	12
	Dyslipidemia	48	0.96	16
	**Depression/mood disorders**	**35**	**3.33**	**54**
	Chronic kidney diseases	29	0.78	13
	**Asthma**	**24**	**3.53**	**57**
	**Thyroid diseases**	**24**	**2.03**	**33**
	Colitis and related diseases	23	1.60	26
	Osteoarthrosis/degenerative joint diseases	23	1.64	27
	**Dorsopathies**	**19**	**2.57**	**42**
	**Osteoporosis**	**17**	**2.25**	**36**
	**Neurotic/stress related/somatoform diseases**	**15**	**4.27**	**69**
	**COPD/emphysema/chronic bronchitis**	**13**	**2.23**	**36**
	**Esophagus/stomach/duodenum diseases**	**11**	**2.21**	**36**

	Obesity	10	0.79	13
	Other MSK and joint diseases	9	1.22	20
	Solid neoplasms	7	0.71	11
	Anemia	6	0.45	7
	**Sleep disorders**	**6**	**2.47**	**40**
	Autoimmune diseases	5	1.03	17
	Inflammatory arthropathies	5	1.20	19
	Deafness/hearing loss	4	0.27	4
	Diabetes	4	0.36	6
	Ischemic heart diseases	4	0.22	4
	Other neurological diseases	4	1.93	31
	Atrial fibrillation	3	0.23	4
	Cerebrovascular diseases	3	0.28	5
	Migraine/facial pain syndrome	3	1.22	20
	Other cardiovascular diseases	3	0.80	13
	Other eye diseases	3	0.59	10
	Cataract/lens diseases	2	0.30	5
	Other genitourinary diseases	2	0.59	10
	Bradycardias/conduction diseases	1	0.60	10
	Cardiac valve diseases	1	0.37	6
	Glaucoma	1	0.16	3
	Other psychiatric/behavioral diseases	1	0.58	9
	Prostate diseases	1	0.33	5
	Blindness/visual loss	0	0.04	1
	Dementia	0	0.00	0
	Heart failure	0	0.00	0
**Sensory impairment/Anemia**	Hypertension	77	1.05	21
	Chronic kidney diseases	62	1.64	32
	Dyslipidemia	41	0.81	16
	**Anemia**	**31**	**2.23**	**44**
	**Deafness/hearing loss**	**27**	**2.07**	**41**
	**Cataract/lens diseases**	**24**	**3.80**	**75**
	**Glaucoma**	**22**	**3.45**	**68**
	**Other eye diseases**	**20**	**3.50**	**69**
	Solid neoplasms	20	1.94	38
	Colitis and related diseases	16	1.08	21
	Ischemic heart diseases	15	0.84	17
	**Blindness/visual loss**	**13**	**2.61**	**51**
	Osteoporosis	12	1.51	30
	Osteoarthrosis/degenerative joint diseases	11	0.79	16
	Cerebrovascular diseases	10	1.16	23
	Diabetes	9	0.87	17
	Thyroid diseases	9	0.76	15
	Dementia	8	0.78	15
	Atrial fibrillation	7	0.64	13
	Autoimmune diseases	6	1.15	23
	Depression/mood disorders	6	0.61	12
	Dorsopathies	6	0.85	17
	Inflammatory arthropathies	6	1.36	27
	Obesity	6	0.45	9
	Other MSK and joint diseases	6	0.84	17
	Prostate diseases	6	1.31	26

	Esophagus/stomach/ duodenum diseases	4	0.74	14
	Other genitourinary diseases	4	1.45	29
	Cardiac valve diseases	3	0.98	19
	Other neurological diseases	3	1.19	23
	COPD/emphysema/chronic bronchitis	2	0.43	8
	Neurotic/stress related/somatoform diseases	2	0.44	9
	Other cardiovascular diseases	2	0.40	8
	Asthma	1	0.20	4
	Heart failure	1	0.07	1
	Migraine/facial pain syndrome	1	0.53	11
	Other psychiatric/behavioral diseases	1	0.55	11
	Bradycardias/conduction diseases	0	0.00	0
	Sleep disorders	0	0.07	1
**Cardiometabolic**	**Heart failure**	**77**	**6.43**	**73**
	Chronic kidney diseases	74	1.95	22
	Hypertension	69	0.95	11
	**Ischemic heart diseases**	**58**	**3.34**	**38**
	**Atrial fibrillation**	**46**	**4.21**	**48**
	Dyslipidemia	44	0.87	10
	**Anemia**	**31**	**2.25**	**25**
	**Diabetes**	**27**	**2.69**	**30**
	**Cerebrovascular diseases**	**20**	**2.22**	**25**
	Osteoarthrosis/degenerative joint dis	20	1.42	16
	Colitis and related diseases	19	1.34	15
	Deafness/hearing loss	19	1.48	17
	**Other cardiovascular diseases**	**19**	**4.84**	**55**
	Obesity	15	1.18	13
	**COPD/emphysema/chronic bronchitis**	**14**	**2.50**	**28**
	**Cardiac valve diseases**	**14**	**5.11**	**58**
	**Bradycardias/conduction diseases**	**13**	**6.27**	**71**
	**Inflammatory arthropathies**	**13**	**2.83**	**32**
	Osteoporosis	12	1.57	18
	Solid neoplasms	12	1.16	13
	Thyroid diseases	12	1.04	12
	Autoimmune diseases	11	2.12	24
	Dorsopathies	11	1.49	17
	Blindness/visual loss	10	1.96	22
	Other MSK and joint diseases	10	1.34	15
	Depression/mood disorders	9	0.89	10
	Other eye diseases	9	1.59	18
	Prostate diseases	9	2.01	23
	Asthma	7	1.05	12
	Cataract lens diseases	7	1.16	13
	Glaucoma	7	1.04	12
	Dementia	6	0.58	7
	Esophagus/stomach/duodenum diseases	6	1.22	14
	Migraine/facial pain syndrome	4	1.63	18
	Other genitourinary diseases	4	1.47	17
	Other psychiatric/behavioral diseases	3	1.07	12
	Sleep disorders	3	1.14	13

	Neurotic/stress related/somatoform diseases	1	0.34	4
	Other neurological diseases	1	0.28	3

Prevalence was calculated as the number of participants with a given disease in each of the patterns divided by the total number of participants in each of the patterns. The O/E ratio was computed by dividing the prevalence of a disease within a specific pattern by its prevalence in the overall multimorbid population. Exclusivity was calculated as the proportion of participants with a given disease within a specific pattern relative to the total number of participants with that disease in the entire multimorbid population.

COPD = chronic obstructive pulmonary disease; MSK = muscle skeletal; O/E = observed/expected ratio.
