# Supplementary material for: Shared and specific blood biomarkers for multimorbidity
Source: Nat Med. 2026 Jan 2;32(2):736–45. doi: 10.1038/s41591-025-04038-2 (PMC12920107; doi:10.1038/s41591-025-04038-2)
Supplement: Supplementary file 2 — Reporting Summary [file 41591_2025_4038_MOESM2_ESM.pdf]

nature portfolio

Corresponding author(s): Davide Liborio Vetrano

Last updated by author(s): Sep 17, 2025

Reporting Summary

Nature Portfolio wishes to improve the reproducibility of the work that we publish. This form provides structure for consistency and transparency in reporting. For further information on Nature Portfolio policies, see our Editorial Policies and the Editorial Policy Checklist.

Statistics

For all statistical analyses, confirm that the following items are present in the figure legend, table legend, main text, or Methods section.

n/a	Confirmed	
☐	☒	The exact sample size (*n*) for each experimental group/condition, given as a discrete number and unit of measurement
☐	☒	A statement on whether measurements were taken from distinct samples or whether the same sample was measured repeatedly
☐	☒	The statistical test(s) used AND whether they are one- or two-sided<br>*Only common tests should be described solely by name; describe more complex techniques in the Methods section.*
☐	☒	A description of all covariates tested
☐	☒	A description of any assumptions or corrections, such as tests of normality and adjustment for multiple comparisons
☐	☒	A full description of the statistical parameters including central tendency (e.g. means) or other basic estimates (e.g. regression coefficient) AND variation (e.g. standard deviation) or associated estimates of uncertainty (e.g. confidence intervals)
☐	☒	For null hypothesis testing, the test statistic (e.g. *F*, *t*, *r*) with confidence intervals, effect sizes, degrees of freedom and *P* value noted<br>*Give P values as exact values whenever suitable.*
☒	☐	For Bayesian analysis, information on the choice of priors and Markov chain Monte Carlo settings
☒	☐	For hierarchical and complex designs, identification of the appropriate level for tests and full reporting of outcomes
☐	☒	Estimates of effect sizes (e.g. Cohen's *d*, Pearson's *r*), indicating how they were calculated

Our web collection on statistics for biologists contains articles on many of the points above.

Software and code

Policy information about availability of computer code

Data collection	SNAC-K data were collected through dedicated interviews and related forms. Information is later electronically read and stored in Stata. The data quality check is regularly carried out following specific and rigorous protocols.<br>BLSA data were collected by study staff following standardized interviews, clinical examinations, and laboratory tests.
Data analysis	All statistical analyses were performed with R version 4.2.3. Specifically, poLCA, glmnet, lme4, factoextra, corrplot, survival, and ggplot2 packages were used for the analysis. BioRender was used for graphical representation.<br>No custom code or mathematical algorithm was employed in the study. Analysis scripts for this study are available at https://github.com/AliRita/Biomarkers-and-Multimorbidity-study.

For manuscripts utilizing custom algorithms or software that are central to the research but not yet described in published literature, software must be made available to editors and reviewers. We strongly encourage code deposition in a community repository (e.g. GitHub). See the Nature Portfolio guidelines for submitting code & software for further information.

Data

Policy information about availability of data

All manuscripts must include a data availability statement. This statement should provide the following information, where applicable:

- Accession codes, unique identifiers, or web links for publicly available datasets
- A description of any restrictions on data availability
- For clinical datasets or third party data, please ensure that the statement adheres to our policy

SNAC-K data are sensitive data; thus, they cannot be shared publicly, but raw and analysed de-identified data can be requested by qualified researchers at https://www.snac-k.se/. The request will be reviewed to ensure confidentiality obligations and intellectual property. A data sharing agreement must be signed prior to data release. BLSA data are availabe to qualified researchers through submission of proposals at https://www.blsa.nih.gov/.

Research involving human participants, their data, or biological material

Policy information about studies with human participants or human data. See also policy information about sex, gender (identity/presentation), and sexual orientation and race, ethnicity and racism.

Reporting on sex and gender	The SNAC-K dataset has information on sex (biological attribute) but not gender.<br>BLSA dataset has information on sex (self-reported biological attribute).<br>Information on sex was reported and used as a covariate throughout the analyses.
Reporting on race, ethnicity, or other socially relevant groupings	SNAC-K cohort is composed of a sample of white individuals 60+ from an affluent area of Stockholm. No other ethnicities are represented in the sample.<br>BLSA sample included 522 individual aged 60+; sample description according to the analysis is reported in Supplementary Table 5.
Population characteristics	In SNAC-K, during the nurse interview, demographic information such as age, sex, and education was obtained.<br>To ensure a comprehensive understanding of participants' health status, a thorough clinical procedure was implemented, as detailed elsewhere. This involved medical history collection during physician interviews, clinical examinations, diagnostic tests (both instrumental and blood tests), and data from inpatient and outpatient records, medical journals, and the Swedish National Patient Register. Diagnoses were coded according to the International Classification of Diseases, 10th revision (ICD-10), following a clinical review conducted by trained physicians. Diagnoses considered chronic were further classified into 60 macro categories.Non-fasting venous blood samples were obtained and serum protein quantification was carried out through several laboratory techniques. Fifty-four serum biomarkers describing four major biological processes (metabolism, inflammation, vascular and neurodegeneration) were available .<br>The BLSA cohort consists of community-dwelling volunteers; for more information, visit the BLSA study website (https://www.blsa.nih.gov/) and reference (https://doi.org/10.1093/geronj/40.6.767)
Recruitment	The study population consists of adults ≥60 years living in the community or in institutions, from the Kungsholmen district of Stockholm, Sweden. A random sample of 11 age cohorts born between 1892 and 1939 (the youngest and oldest age cohorts were oversampled) was invited to participate in the study. People who agreed to participate were evaluated for the first time between 2001 and 2004. Participants who were <78 years of age were then followed up every six years and participants ≥78 years every three years. At baseline, 3363 people were examined (participation rate 73%). Non-participants were older, more likely females, and more likely institutionalized. As a consequence, non-participants may have presented a higher disease burden. A participation rate of 73% remains among the highest reported in population-based studies involving older people. The present study is based on data collected at baseline and at each follow-up for up to 12 years.<br>In BLSA, community-dwelling volunteers were recruited from the city and surrounding areas of Baltimore, Maryland. Participants were then followed up with an age-dependent frequency (<60 every 4 years, 60-79 every 2 year, >80 every year) to account for the faster functional decline in the later part of the life.
Ethics oversight	The research protocol for each phase of the SNAC-K study received approval from the Regional Ethical Review Board in Stockholm, and ethical standards of the Declaration of Helsinki were followed throughout the investigation.<br>The BLSA study was approved by the National Institutes of Health Intramural Research Program Institutional Review Board, and informed consent was obtained from each participant. Deidentified data were used for analyses.

Note that full information on the approval of the study protocol must also be provided in the manuscript.

Field-specific reporting

Please select the one below that is the best fit for your research. If you are not sure, read the appropriate sections before making your selection.

☒ Life sciences ☐ Behavioural & social sciences ☐ Ecological, evolutionary & environmental sciences

For a reference copy of the document with all sections, see nature.com/documents/nr-reporting-summary-flat.pdf

Life sciences study design

All studies must disclose on these points even when the disclosure is negative.

Sample size	No sample size calculations have been carried out. The present study is based on the population-based study SNAC-K, which includes at baseline 3363 individuals aged 60+. SNAC-K is one of the biggest population-based studies on aging. This size allows to study most of the age-related conditions with decent statistical power, including multimorbidity.<br>In BLSA, sample size is not predetermined. We included individuals with availble data on key demographic characteristics, blood-based biomarkers, and chronic conditions at baseline.
Data exclusions	From the initial cohort of SNAC-K participants at baseline (n=3,363), we excluded individuals with at least one blood-based biomarker measurement missing (n = 1116), obtaining a final analytical sample of 2247 individuals. Participants with missing biomarker data were older, more likely to be female, less educated, and had a higher burden of chronic diseases compared to those with complete data. T<br>his study incorporated data from BLSA participants' first assessment with complete information through a maximum follow-up duration of 15 years. Individuals aged 60 years or older with available data on baseline blood-based biomarkers, key demographic characteristics, and chronic conditions were included, obtaining a final study sample of 522 participants. This subset of BLSA participants is, on average, slightly older than other participants aged 60 years or older observed during the same period (August 7, 2006 – March 4, 2025).
Replication	The rigorous disease clinical assessment, the representativeness of the sample population (response rate 73%, among the highest in this field), the wide panel of biomarkers, and the use of clear statistical methods ease the full replicability of the present study. However, we were able to perform an external validation of our longitudinal findings in a sample from the BLSA study
Randomization	Randomization was not aplicable to our study design.
Blinding	NA

Reporting for specific materials, systems and methods

We require information from authors about some types of materials, experimental systems and methods used in many studies. Here, indicate whether each material, system or method listed is relevant to your study. If you are not sure if a list item applies to your research, read the appropriate section before selecting a response.

Materials & experimental systems

n/a	Involved in the study	
☐	☒	Antibodies
☒	☐	Eukaryotic cell lines
☒	☐	Palaeontology and archaeology
☒	☐	Animals and other organisms
☐	☒	Clinical data
☒	☐	Dual use research of concern
☒	☐	Plants

Methods

n/a	Involved in the study	
☒	☐	ChIP-seq
☒	☐	Flow cytometry
☒	☐	MRI-based neuroimaging

Antibodies

Antibodies used	In SNAC-K, the serum concentrations of NfL and GFAP were measured using Simoa Neuro 2-plex B Kit (Quanterix, product number 103520 and Lot# 503409). Simoa Neuro 3-plex A Kit (Quanterix, product number 101995 and Lot# 503659) was used to measure serum Aβ40, Aβ42 and t-tau and Simoa pTau-181 Advantage V2 Kit (Quanterix, product number 103714 and Lot# 503703) was used to measure serum p-tau181. For each kit, 25 µL of sample were diluted 1:4 and the assays were performed according to manufacturer instructions. The Quanterix instrument provides, for all proteins, AEB (average enzyme per bead) values for calibrators, controls and samples. The Quanterix SR-X software automatically performs curve-fitting, extrapolation of concentrations and graphical representation using the calibrators, a series of known concentration of an analyte, and a four-parameter logistic (4PL) curve fit. Baseline interleukin (IL-1beta, IL-6, IL-8, IL-10, IL-12p70), INF-gamma, and TNF-alpha were measured at Accelerator Laboratory Services, Quanterix Corp., in Billerica (MA, USA), using Simoa CorPlex Human Cytokine Panel 1 on the Quanterix® SP-XTM imaging and analysis platform.<br>In BLSA, fasting insulin and leptin are measured in the IRP-NIA Laboratory of Clinical Investigation by enzyme-linked immunosorbent assay (ELISA) (ALPCO, Salem, NH, USA; Linco Research, Inc., St. Charles, MO, USA). GDF15 and Cystatin C were measured using the 7k SomaScan assay v4.1 (SomaLogic, Inc.; Boulder, CO, USA).
Validation	https://www.quanterix.com/wp-content/uploads/2022/10/pTau-181-Advantage-V2.1-HD-1-HD-X-Data-Sheet.pdf for p-tau 181<br>https://www.quanterix.com/wp-content/uploads/2020/12/N2PB_SR-X_Data_Sheet_rev01.pdf for NfL and GFAP<br>https://www.quanterix.com/wp-content/uploads/2020/12/Simoa_N3PA_Data_Sheet-SR-X_0.pdf for abeta and total tau

Clinical data

Policy information about clinical studies

All manuscripts should comply with the ICMJE guidelines for publication of clinical research and a completed CONSORT checklist must be included with all submissions.

Clinical trial registration	NA
Study protocol	Not available. The present study is part of a project funded by the Swedish Research Council.
Data collection	SNAC-K data collection (including clinical, functional, psychological examinations and laboratory testing) are carried out in a dedicated research center by trained physicians, nurses and neuro-psychologists. The procedure is carried out in a friendly and calm environment. People who agreed to participate were evaluated for the first time between 2001 and 2004. Each assessment lasts between 4 and 5 hours.<br>In BLSA, enrolled participants are followed up with an age-dependent frequency (<60 every 4 years, 60-79 every 2 year, >80 every year) to account for the faster functional decline in the later part of the life. All assessments were performed by trained and certified staff following standardized protocols.
Outcomes	In SNAC-K, diseases were identified at each visit through medical history, physical examination, and in-person and/or proxy interviews. Additional diseases were identified based on laboratory parameters, medication usage, and data from inpatient and outpatient records, medical journals, and the Swedish National Patient Register. These diagnoses were subsequently coded according to the 10th revision of the International Classification of Diseases (ICD-10). An international team of physicians and epidemiologists classified diseases as chronic when they persisted over time and were associated with i) ongoing disability or reduced quality of life ii) the need for prolonged care, treatment, or rehabilitation. The diseases identified as chronic were then grouped into 60 broad categories, which were used to define the different measures of multimorbidity.<br>First, multimorbidity was defined as the number of chronic conditions, both at cross-sectional and longitudinal levels. Then, chronic diseases with a prevalence of at least 2% were used to identify - throught latent class analysis - homogeneous groups of individuals with multimorbidity (i.e., 2+ diseases) sharing similar patterns of chronic diseases. Multimorbidity patterns were labeled based on the overexpressed diseases (i.e., those with an observed/expected ratio ≥ 2 and an exclusivity of ≥25%). Participants were assigned to the pattern with the highest probability of membership.<br>In BLSA, diseases were identified based on a combination of clinical observations during comprehensive physical examination, self-reported medical history, clinical laboratory parameters, and medication use. Where possible classifications were made based upon the 9th revision of the ICD (ICD-9) codes, and medications were coded according to the Anatomical Therapeutic Chemical (ATC) system.

Plants

Seed stocks	NA
Novel plant genotypes	NA
Authentication	NA
